# Predicting financial market crashes using ghost singularities

**DOI:** 10.1371/journal.pone.0195265

**Published:** 2018-03-29

**Authors:** Damian Smug, Peter Ashwin, Didier Sornette

**Affiliations:** 1 Centre for Systems, Dynamics and Control, Department of Mathematics, Harrison Building, University of Exeter, Exeter EX4 4QF, United Kingdom; 2 ETH Zürich, Department of Management, Technology and Economics, Scheuchzerstrasse 7, CH-8092 Zürich, Switzerland; 3 Swiss Finance Institute, c/o University of Geneva, 40 blvd. Du Pont d’Arve, CH 1211 Geneva 4, Switzerland; Polish Academy of Sciences, Institute of Nuclear Physics, POLAND

## Abstract

We analyse the behaviour of a non-linear model of coupled stock and bond prices exhibiting periodically collapsing bubbles. By using the formalism of dynamical system theory, we explain what drives the bubbles and how foreshocks or aftershocks are generated. A dynamical phase space representation of that system coupled with standard multiplicative noise rationalises the log-periodic power law singularity pattern documented in many historical financial bubbles. The notion of ‘ghosts of finite-time singularities’ is introduced and used to estimate the end of an evolving bubble, using finite-time singularities of an approximate normal form near the bifurcation point. We test the forecasting skill of this method on different stochastic price realisations and compare with Monte Carlo simulations of the full system. Remarkably, the approximate normal form is significantly more precise and less biased. Moreover, the method of ghosts of singularities is less sensitive to the noise realisation, thus providing more robust forecasts.

## Introduction

Forecasting market behaviour has been a topic of general interest for hundreds of years. At the same time, given the complexity of financial markets, mathematical models have been limited to explore in a rather fragmented way some of the many mechanisms and dynamics at play in the real world. For instance, there are models exploring the impact of the dynamics between traders (see e.g. [1–6]), other models attempt to capture the effects of feedbacks between financial information and investment strategies using various stochastic non-linear processes (see e.g. [7–10]). These references can be conceptually linked to the pioneering work of [11–16] describing the dynamical behaviour of heterogeneous markets with many trader types using dynamical system concepts, including limit cycles as the large type limit of interaction agents, bifurcation routes to instability and strange attractors in evolutionary financial market models. This variety suggests that the concepts and methods of complex dynamical systems could be useful in the area of financial markets. A certain type of non-linearity is of particular interest due to its large impact in generating what are arguably the most visible deviations from normally (quasi-)efficient financial markets, namely financial bubbles. During financial bubbles, positive feedback mechanisms give rise to the so-called super-exponential acceleration of prices [17–20], followed by the burst of the bubble in large drawdowns [21–23], i.e. crashes. A parsimonious representation of this super-exponential dynamics takes the form of finite-time singularity models [24–28].

In the present paper, we revisit the model of coupled stock and bond prices introduced by [10], which exhibits periodically collapsing bubbles in a certain domain of the parameter space. We extract the underlying finite-time singularities and use the associated trajectories to develop a method to predict the bubble collapses. First, we develop our formalism and technique to provide credible predictions of the stock price falls in the deterministic case. We then test the method in a stochastic extension of the model. Using the theory of dynamical systems, we introduce the notion of *ghosts of finite-time singularities*.

The concept of ghosts of singularities emerges from studying stable periodic solutions in the neighbourhood of a saddle-node bifurcation. We realised that, even if the trajectory close to the saddle-node point can be well approximated by a truncated normal form, further away, the differences might become infinitely large (for instance, if the periodic orbit stays bounded and the normal form approximation exhibits a blow up in finite time), but the period can still be well estimated. In general, we will use the notion of ghosts of finite-time singularities to describe the rapid changes in periodic solutions, for which the truncated normal form leads to *real* finite-time singularities that are not observable in the original system. A key insight is that, even if we cannot approximate the exact shape of the periodic function, we are able to obtain another piece of information that is almost as important—its periodicity. We show that the knowledge about the period of oscillations can indeed help forecast the time of a crash.

The article is constructed as follows. In the next section we explain the construction of the model we investigate, build basic intuitions on the bubbles and crashes for the deterministic and stochastic versions and justify dynamically several stylized facts observed in financial markets. We then introduce the notion of a ghost of a finite-time singularity for a simple non-linear ODE and test its predictive power on the deterministic system of coupled stocks and bonds. Moreover, we present the comparison of the predictive performance of our methodology compared with the standard unconditional Monte Carlo approach in the stochastically extended system. The conclusion section summarises our main results.

## Non-linear dynamical system of stocks and bonds

In this section, we recall the dynamical system of [10] and provide dynamical explanations for a variety of market events. We also point out the existing bifurcations and how the system responds to parameter shifts. The latter will be quantified in the neighbourhood of bifurcation lines. The system is extended into a version with multiplicative noise to model stochastic price fluctuations.

### Definition of the dynamical system

A dynamical system of a coupled pair of one bond and one asset is introduced in [10]:

$$\begin{array}{c} \left\{ \begin{array}{c} \dot{x}=x-x^{2}\cdot e^{-bxz}\\ \dot{z}=z-z^{2}\cdot e^{-gx} \end{array}\;.\right. \end{array}$$ (1)

The system is designed from a self-financing portfolio and links the price of an asset/stock (*x*) with the price of a bond (*z*), the latter quantifying the cost of borrowing. As can be observed later, the amplitude of variations of *z* is too large to be directly interpreted as a real bond price and one can treat *z* as the investors confidence for further growth of stock market. Although qualitatively the bond price is positively correlated with the confidence, for this paper we keep to an analysis of the model presented in [10] and leave its generalization to future work.

**Fig 1:**
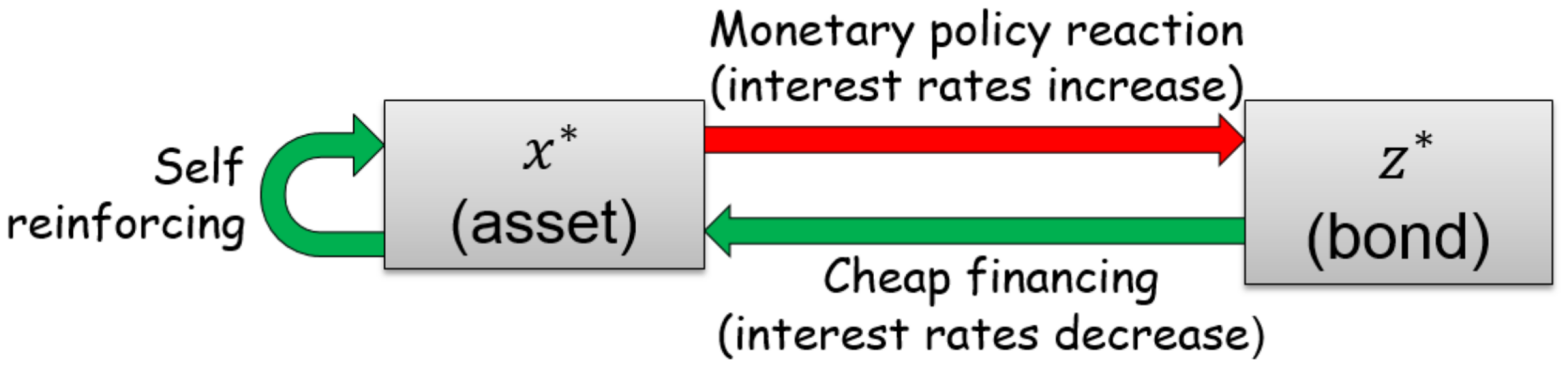
Schematic diagram showing the feedback loops governing the prices *x*(*t*) and *z*(*t*) for the model (1) of [10]. An increasing bond price means mechanically a lower interest rate and thus a lower borrowing cost, which favours further stock price increase (term *e*^−*bxz*^ with *b* > 0). A large stock price leads to a reaction of the central bank to increase the interest rates and thus decrease the bond price (term *e*^−*gx*^ with *g* < 0). A larger stock price is also assumed to feedback positively on itself (term *e*^−*bxz*^ with *b* > 0).

Parameter *b* > 0 stands for the sensitivity of the fundamental asset price on past asset and bond prices, and parameter *g* < 0 is the sensitivity of the fundamental bond price to past asset prices. The scheme of feedback loops governing the equilibria of these two variables is presented in Fig 1 and is more thoroughly explained by [10]. One can think of the terms *e*^−*bxz*^ and *e*^−*gx*^ as quantifying the amplitudes of the forces that tend to push the prices back to their fundamental values. Depending on the parameters in system (1), three different scenarios can be observed for the same initial conditions: convergence to a stable fixed point, divergence to infinity (only for *g* > 0) or convergence to a stable periodic orbit. In the following, we classify the different bifurcations exhibited by system (1), making more precise and extending the analysis of [10]. We shall focus on the non-linear periodic orbits as their properties make them reasonable candidates to represent bubbles and crashes in real financial markets.

#### Definition of bubbles

We shall call ‘bubble’ each transient part of a periodic orbit during which the price *x* accelerates in a super-exponential fashion, and which is followed by a fast correction. In the deterministic version (1) of the model, these bubble regimes occur only for certain periodic orbits. The bubbles are thus occurring periodically, hence the title of ‘periodically collapsing bubbles’ in [10].

### Bifurcations of fixed points and periodic orbits

**Fig 2:**
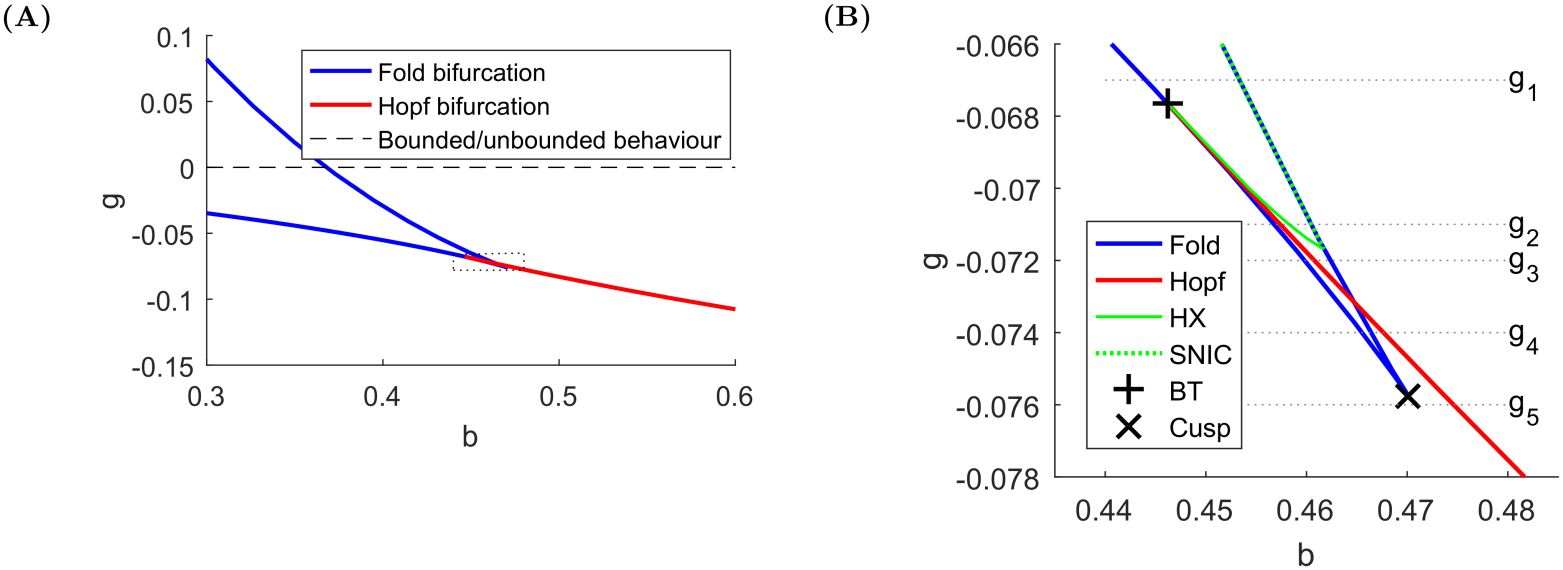
Two-parameter bifurcation diagram. **(A)** Macroscopic view, **(B)** zoom-in on the marked rectangle in Panel (A). The dotted lines represent certain bifurcation paths, which are shown in Fig 3. BT means Bogdanov-Takens point. Bifurcations are computed using XPPAUT [31].

In a macroscopic view of the two parameter plane for the system (1), one can observe two lines of bifurcations of fixed points—saddle-node and Hopf [10], which are presented in Fig 2A. However, taking a closer look at the region where these two lines meet, it turns out that it is not a single point, but a set of points where bifurcations of codimension-two occur. From an economic point of view, this means that a very small change in the market conditions can change the system from a stable one to one that exhibits regular crashes. Of course, these boundaries between different regimes will be smoothed out somewhat when a stochastic component is added (see e.g. [29]), as we also show below. S1 Appendix provides the exact values for the points where bifurcation lines meet (Bogdanov-Takens and cusp points). After tracking certain bifurcation paths varying *b* with fixed *g*, one can see that the bubbles are not necessarily born from a Hopf bifurcation, but arise after ‘having’ at first an infinite period. Two cases must thus be considered:

1. The saddle is always present. The periodic orbit grows until it hits the saddle and the homoclinic connecting orbit appears (see Fig 8.12. in [30]). For one parameter value, the stable and unstable separatrix of the saddle are connected. To refer to this situation, we will use the abbreviation **HX**.
2. The other possibility is that a periodic orbit is born from a collision of a saddle and a node, which makes both equilibria disappear. The set of parameters for which the collisions occur forms a saddle-node invariant circle (denoted **SNIC**).

The **HX** bifurcation line was tracked in the two-parameter plane by following an orbit of large period, whereas the **SNIC** coincides with a saddle-node branch. In Fig 2B, both lines are indicated.

**Fig 3:**
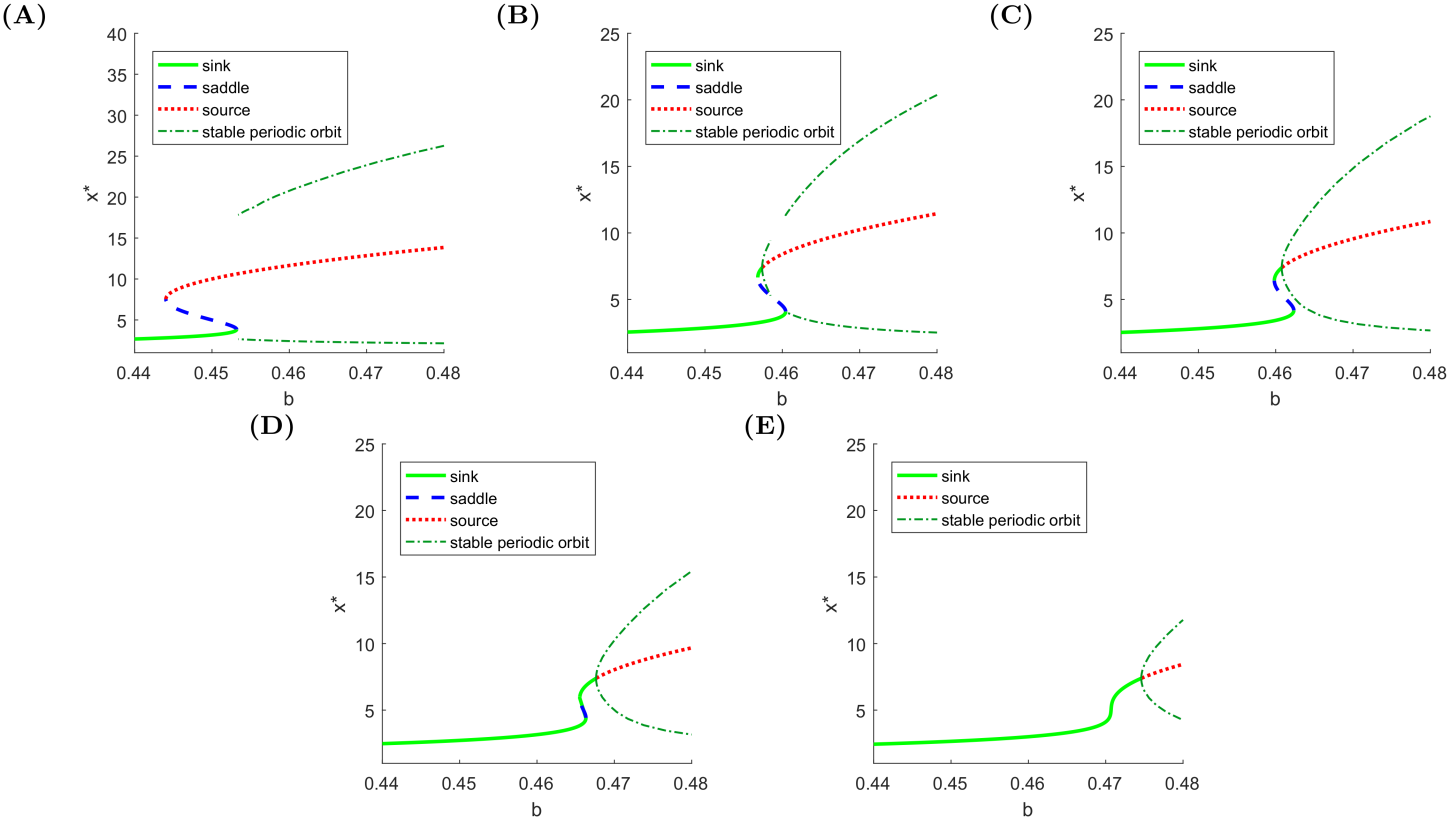
One parameter bifurcation diagrams for fixed *g* < 0. **(A)** *g* = −0.067, periodic orbits appear when a saddle collides with a sink, **(B)** *g* = −0.071, periodic orbits appear via a Hopf bifurcation, exist until a homoclinic bifurcation occurs and then appear again when a saddle collides with a sink, **(C)** *g* = −0.072, periodic orbits emerge from a Hopf bifurcation point, which occurs between two fold bifurcations, **(D)** *g* = −0.074, the fold bifurcations are approaching each other, **(E)** *g* = −0.076, only the Hopf bifurcation remains, from which periodic orbits appear.

In order to show how changes of parameter affect the stability of the fixed points and of the periodic orbits, Fig 3 presents a set of bifurcation diagrams where chosen paths for different values of *g* are shown to determine the bifurcation order. The selected parameter values exhaust all the possible situations. Note that the periodic orbits always emerge if either a saddle collides with a stable node or via a Hopf bifurcation.

### Analysis of scaling laws governing the period and amplitude of the bubbles

A numerical study of the period and amplitude of bubbles in the vicinity of certain singularity lines is presented in [10]. Here, we present analytical validations of those results.

#### Period of the bubbles

In the bifurcation diagram 3B, there are two disjoint intervals where bubbles occur. As defined before, a necessary condition for a bubble to occur is the presence of a periodic orbit (in the deterministic version (1) of the dynamical system). We find that oscillations are born in Hopf bifurcation and remain until a HX bifurcation, then they appear again in a SNIC. These two situations are governed by different scaling laws:

1. approaching HX (see [32]):
  $$\begin{array}{c} f_{hx}\left(b\right)=c\cdot \ln\left|b-b_{hx}\right|\;, \end{array}$$ (2)
2. approaching SNIC (Example 4.3.1 in [33]):
  $$\begin{array}{c} f_{snic}\left(b\right)=c\cdot {\left|b-b_{snic}\right|}^{-1/2}\;. \end{array}$$ (3)

**Fig 4:**
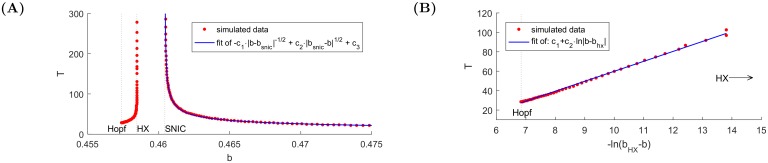
Analysis of the orbit periods for *g*_2_ = −0.071. **(A)** Scenario 1 of approaching HX (left series of dots) and scenario 2 of approaching SNIC (right series of dots fitted by expression (3)), **(B)** Scenario 1 of approaching HX: logarithmic transformation of the distance from the bifurcation point approximated by a linear function.

In the second case, adding higher order terms may give better fits. Fig 4 shows good agreements with the best fits of these functions using the *L*^2^ norm.

#### Amplitude

In S2 Appendix, we provide a short analytical study proving that the amplitude of bubbles for *g* → 0^−^ is governed by a scaling proportional to 1/|*g*|, which supports the numerical results of [10]. The main argument relies on the scaling law 1/|*g*| governing the position of the saddle fixed point, which provides a lower bound for the amplitude of the peak of a bubble. Furthermore, for *g* → 0^−^, the ratio of the bubble amplitude to the position of the saddle appears to converge to a constant between 1 and 2, which suggests the same scaling law holds for the bubble amplitude.

### Stochastic dynamical system of stocks and bonds

In order to investigate the extent to which the classification of the different regimes of the deterministic system (1) informs us on the behaviours of prices in the presence of a stochastic component, and how the concept of ghosts of finite-time singularities presented below applies to the noisy situation, we first present numerical illustrations of a stochastic differential equations (SDE) version of (1) with multiplicative noise. Specifically, we study the following SDEs

$$\begin{array}{c} \left\{ \begin{array}{c} dx=\left(x-x^{2}\cdot e^{-bxz}\right)dt+\sigma_{x}\;x\;dW_{t}^{\left(1\right)}\\ dz=\left(z-z^{2}\cdot e^{-gx}\right)dt+\sigma_{z}\;z\;dW_{t}^{\left(2\right)}. \end{array}\right. \end{array}$$ (4)

The two new terms $\sigma_{x}\;x\;dW_{t}^{\left(1\right)}$ and $\sigma_{z}\;z\;dW_{t}^{\left(2\right)}$ correspond to the standard multiplicative proportional stochastic components of financial price models. For *b*, *g* → +∞, the two non-linear terms vanish, and the system (4) reduces to two standard geometric Brownian motions (GBM). Nevertheless, in the system we analyse, the natural value of *g* resides in the interval (−∞, 0). Making |*g*| small (*g* → 0^−^) corresponds to decreasing the sensitivity of bond price on stocks. Further growth of *g* above 0 means that the sensitivity is actually inverted and *g* → +∞ should be understood as not large sensitivity, but an inverted way with which the stocks have an impact on the bonds. Moreover, *g* → +∞ ⇒ *z* → +∞ which implies that *e*^−*bxz*^ → 0. Then, for larger times the stock price decouples from the bond price and even for small fixed positive values of *b* the GBM for *x* can be recovered. It is important to notice, that both *b* → ∞ and *b* = 0 lead to such decoupling, but in the latter case *x* is bounded since the deterministic part is a pure logistic equation in *x*. The *z* dependence for *b* > 0 thus amounts to decreasing the impact of the bound, in other words, increases the bound for the price *x*.

Here, the volatilities *σ*_*x*_ and *σ*_*z*_ are constant and the two Wiener processes $dW_{t}^{\left(1\right)}$ and $dW_{t}^{\left(2\right)}$ are correlated with a constant correlation coefficient of 0.5. This value is justified as, according to [34], correlations between postwar returns in stock and bond prices were around 0.4 in the U.S. and around 0.6 in the U.K. However, during bubbles and crashes, correlations tend to vary a lot [35–37], hence it would be interesting to investigate the impact of regime switches in the amplitude of the correlation coefficient. We leave this for a future work.

In our simulations, we analyse the stochastic dynamics close to the saddle-node bifurcation at an arbitrarily chosen point *b* = 0.42 and *g* = −0.04. We use the empirical evidence that the daily standard deviation for stock prices (resp. bond prices) is of the order of a few percent, say 3% (resp. a few basis point, i.e. a few hundreds of a percent, say 0.05%). Taking the reduced time unit of our model to correspond to approximately three months of the real world, this yields that reasonable values of *σ*_*x*_ should lie in the interval [0.2, 0.6] and of *σ*_*z*_ in the interval [0, 0.01].

The values of *σ*_*x*_ could be retrieved in another way as well. Assuming that the typical standard deviation in the GBM model of stock prices with daily time units is approximately of order 10^−4^ (say 0.0004) and one time unit in our model is around 100 days, rescaling the variance proportionally to the time we obtain the new value of $\bar{\sigma }=\sqrt{100}\cdot 0.0004=0.004$ and the GBM can be written in the new time units as $dp=\mu pdt+\bar{\sigma }pdW_{t}$. If we take the drift coefficient *μ* to be of order 1% in 100 days (around 3% in a year), we obtain *dp* = 0.01 ⋅ *pdt* + 0.004 ⋅ *pdW*_*t*_ and the proportion for stock price $\frac{\bar{\sigma }}{\mu }=0.4$ is the same as in the model (4) with $\frac{\sigma_{x}}{1}=0.4$.

We leave for another work the problem of a rigorous calibration of the model to real data, as it will require specially adapted maximum likelihood methods, generalised methods of moments and/or Kalman filtering.

**Fig 5:**
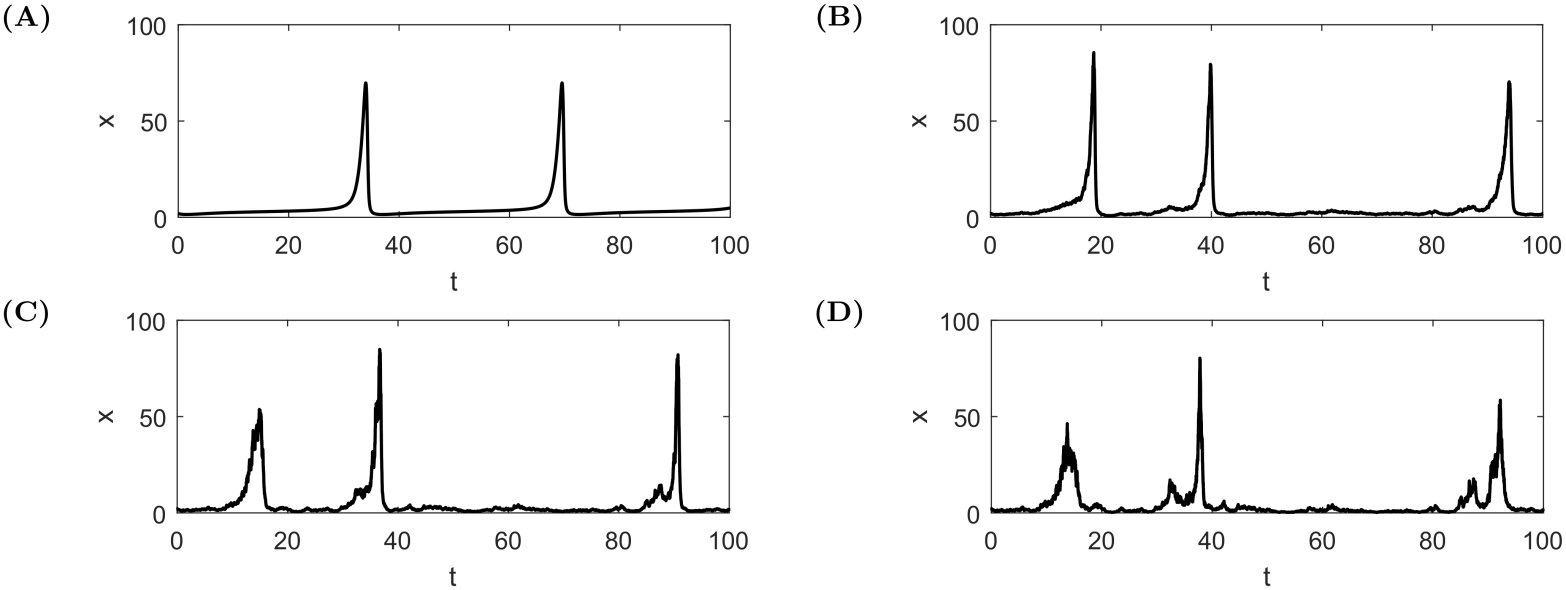
Sample bubbles generated with system (4) with various noise level for the same realisations of the random increments $dW_{t}^{\left(1\right)}$ and $dW_{t}^{\left(2\right)}$. The parameters are *b* = 0.42, *g* = −0.04, $\text{Corr}(W_{t}^{\left(1\right)},W_{t}^{\left(2\right)})=50\%$, initial conditions: *x*_0_ = 2, *z*_0_ = 0.3, **(A)** *σ*_*x*_ = 0.0, *σ*_*z*_ = 0.00, **(B)** *σ*_*x*_ = 0.2, *σ*_*z*_ = 0.01, **(C)** *σ*_*x*_ = 0.4, *σ*_*z*_ = 0.01, **(D)** *σ*_*x*_ = 0.6, *σ*_*z*_ = 0.01.

Fig 5 shows four trajectories obtained by numerical integration of (4) for different levels of noise (different values of *σ*_*x*_ and *σ*_*z*_). The first observation is that the stochastic system also exhibits recurring bubbles with qualitatively similar shapes to the deterministic case. However, rather than being precisely periodic, one can observe some variability in the waiting times between them. This can be rationalised by viewing stochastic innovations as providing effective changes of initial conditions along the price paths. Stochasticity also introduces randomness in the amplitude of the bubbles, some being smaller and others larger than in the deterministic periodic case. Another interesting observation is that, as the noise amplitude increases, bubbles are accompanied by ‘foreshocks’ and ‘aftershocks’, namely significant price activities before and after a main price peak. These qualitative observations will be explained thoroughly in the next section.

### Dynamical bubble—Genesis

**Fig 6:**
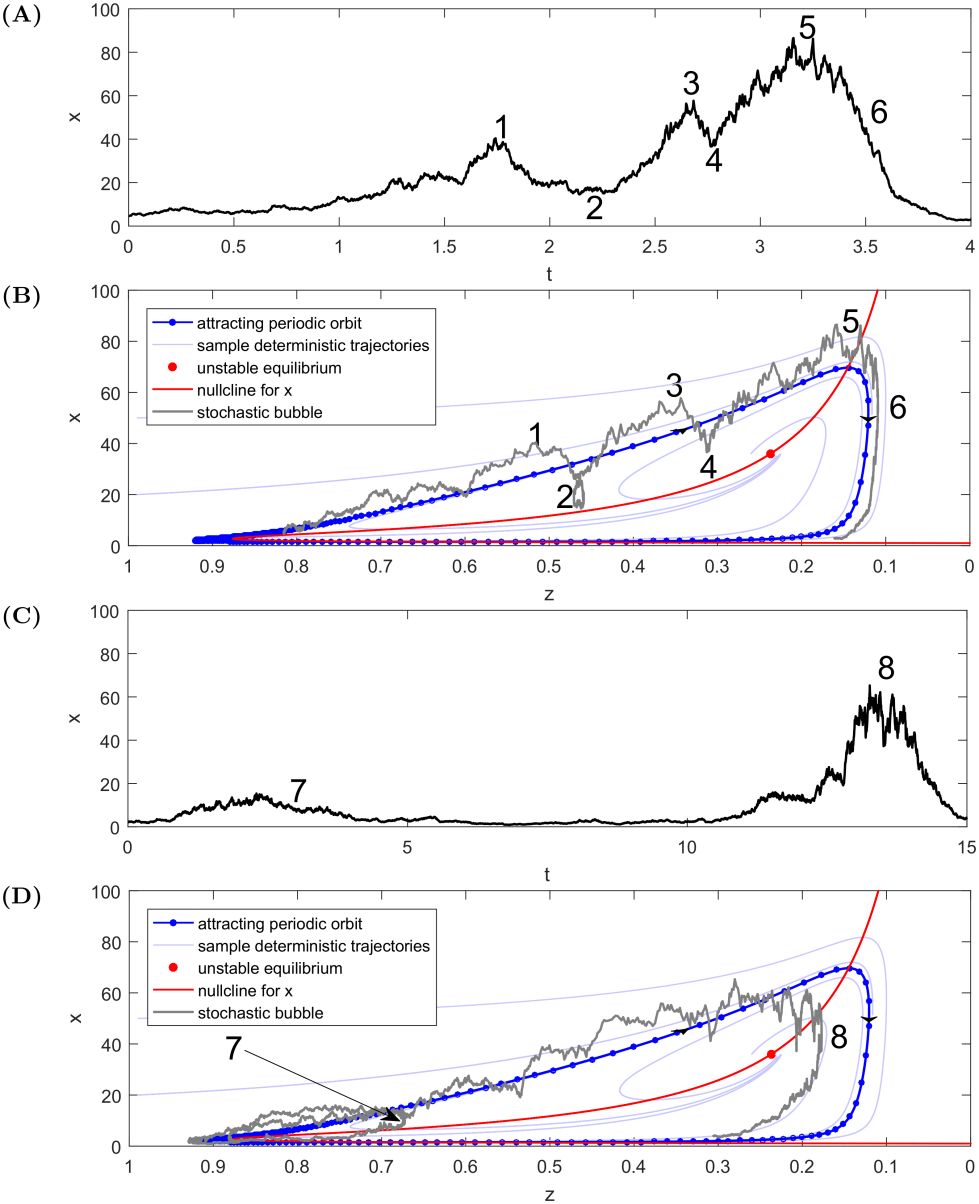
Schematic representation showing different phases and outcomes of stochastic bubbles for (4) with *b* = 0.42, *g* = −0.04, *σ*_*x*_ = 0.6, *σ*_*z*_ = 0.0. The bold blue line represents a deterministic stable orbit with blue dots at fixed time intervals. **(A)** and **(B)** Foreshocks {1/2} and {3/4} preceding the main crash {5/6} are clearly visible. The time intervals between the consecutive local peaks and corrections decrease as a result of at least two factors: first, until around *z* = 0.2, the price *x* accelerates making it prone to take a time consuming detour for smaller *x* {2} that becomes less impactful compared to when *x* is larger {4}. **(C)** and **(D)** The noise can push the dynamics to enter the region where deterministic forces drag trajectories back to the region of small *x*, thus aborting the bubble {7} before it fully develops. The same kind of behaviour for higher *x* levels can shorten the life of a bubble in the regime close to the unstable fixed point where the price dynamics can oscillate temporarily before the main crash {8}.

In order to rationalise the qualitative properties illustrated in Fig 5, let us study an arising bubble in phase space instead of in the time domain. The way a bubble grows or deflates can be driven by a variety of forces that can be investigated conveniently in phase space. The phase diagrams of Fig 6 present the crucial drivers of bubbles, which are now listed.

- **Nullclines** (the curves where either $\dot{x}=0$ or $\dot{z}=0$) and their intersection—unstable fixed point. As the nullcline for *x* (red curve) is the most important for the stock price dynamics, to ensure the clarity of the graphical representation, the nullcline for *z* is not included. When the trajectory lies close to the nullcline for *x*, the dynamics of the stock price becomes almost entirely driven by the stochastic component. Close to the equilibrium fixed point (red dot) or their ghosts (*x* ≈ 0, *z* ≈ 0.9), the deterministic trend of the bond price disappears as well.
- **Deterministic bubble path** to which the stochastic trajectories are attracted (blue bold curve). The system evolves clockwise and the density of dots represents the inverse of the speed of the representative point (one dot is plotted every fixed time period). For small *x*, the price changes happen at a much slower pace than for higher values of *x* and the largest speed is reached when *x* collapses in what can be termed as a crash.
- **Other sample deterministic trajectories** help understanding the future evolution of the system. Every trajectory spirals clockwise, some of them prematurely abort the bubble, whereas other trajectories approach the bubble much further from the equilibrium ghost point and will be instantly directed to take the loop around.

These terms are useful in explaining what really drives the development of each specific bubble. In the presence of noise, there are various patterns occurring in the system, which in turn capture several stylized facts observed in real financial markets. Let us focus on the three main stages of the development of a bubble. The numbers in curly brackets stand for certain situations presented in Fig 6.

#### Foreshocks

When a bubble begins, stochastic fluctuations can push the trajectory above or below its underlying deterministic version. If *x* happens to be pushed down intensively in a small time period, the dynamics may be driven back and terminate the bubble, leading to the abortion of the main loop—see point {7} in Fig 6C and 6D. The shape of the deterministic vector field shows that, for smaller values of *x*, the burgeoning bubble trajectory can be pushed back to the ghost fixed point (*x* = 0, *z* = 0.9) with relatively smaller levels of noise (the bubble aborts). For other trajectories tracking the deterministic bold blue line, much larger levels of noise are needed to push the price along such a detour, unless the system approaches the end of bubble or the unstable equilibrium when the deterministic force will gradually make *x* decrease.

On the other hand, the dynamics of *x* can withdraw partially, spiral out and create another bubble again {2}. The latter situation can occur several times {2, 4} before the main crash {6} happens. Moreover, the higher the level of *x*, the quicker the progression along the underlying deterministic bubble path. As *x* increases, the price trajectory becomes influenced by underlying deterministic trajectories spiralling out that changes in shape, compared to the phase space region for smaller *x*’s. The higher *x* is, the smaller can be a partial detour away from the deterministic trajectory. As the noise is multiplicative, the same Wiener increments give larger variations of *x* as the dynamics evolves, so that more structures can be observed along the bubble growth. Faster evolution leads to a decrease in the time intervals between the consecutive price peaks. When price peaks are observed in phase space for *x* = 10, *x* = 20 and *x* = 30, in time space the latter two will be closer to each other than the first two.

**Fig 7:**
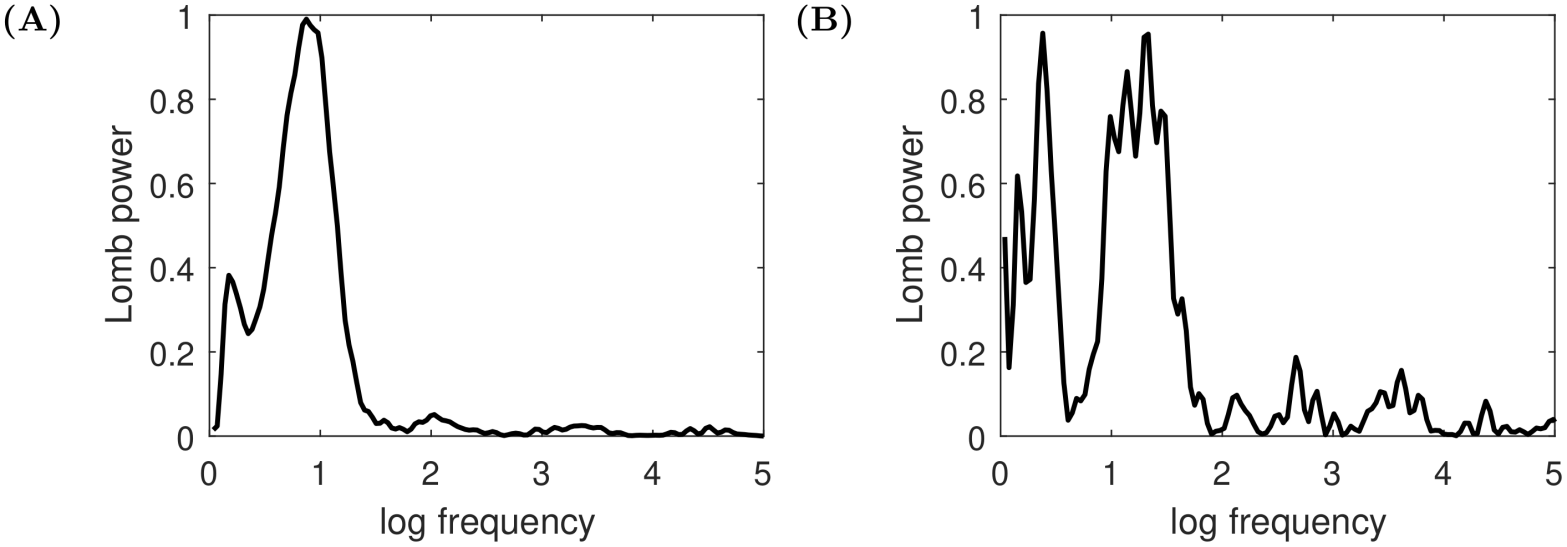
Lomb-periodograms for the trajectories presented in Fig 6. The selection of *t*_*c*_ is done through scanning different possible values between the maximum of the timeseries until the end of sample and then by picking the one giving the highest peak in the Lomb-periodogram. **(A)** The optimised *t*_*c*_ = 3.46, which is in the middle of the deflating bubble, **(B)** the optimised *t*_*c*_ = 13.26, which is at the top of the bubble. In the second case the sample was shortened so it begins at time 11.5, when the bubble starts to develop.

All those factors add up and lead to the birth of smaller structures with accelerating periodicity preceding the end of the bubble and the start of the main crash. These patterns are reminiscent of those observed in real financial bubbles, in particular the joint acceleration of price and of price oscillations captured by the log-periodic power law singularity model [24, 38–40]. To support this observation we produce Lomb-periodograms (Fig 7) of the trajectories presented in Fig 6 (for more information on log-periodicity see [41–43]). The spectral analysis is performed on the residuals *R*(*t*) of the logarithmic series ln(*x*(*t*)) as a function of the variable ln(*t*_*c*_ − *t*) optimised for the highest peak in the Lomb-periodogram with respect to *t*_*c*_. The residuals are obtained through a transformation given in Eq (18) from [38]:

$$\begin{array}{c} R\left(t\right)=\frac{\ln\left(x\left(t\right)\right)-A-B{\left(t_{c}-t\right)}^{\beta }}{C{\left(t_{c}-t\right)}^{\beta }} \end{array}$$ (5)

with *A* = 10, *B* = 1.0, *C* = 0.76, *β* = 0.10 for the series presented in Fig 7A and *A* = 4.5, *B* = 1.0, *C* = 0.34, *β* = 0.44 for the series presented in Fig 7B. In both diagrams one can spot a clear peak characterising the most common log frequencies which suggests the presence of log-periodicity.

The peaks presented in Fig 7 at frequencies around 0.1 − 0.3 correspond to remains of the slow trend over the whole time interval of analysis. The peaks at *f*_1_ ≈ 0.9 in Fig 7A and *f*_2_ ≈ 1.3 in Fig 7B are the signal associated with genuine log-periodicity. The corresponding angular frequencies are *ω*_1_ = 2*πf*_1_ ≈ 5.7 and *ω*_2_ = 2*πf*_2_ ≈ 8.2, whereas the preferred scaling ratios are $\lambda_{1}:=e^{1/f_{1}}\approx 3.0$ in the first case and $\lambda_{2}:=e^{1/f_{2}}\approx 2.2$ in the second one. λ_1_ and λ_2_ quantify the ratio between the shrinking intervals defined by successive price peaks. The results are not sensitive to the method for selecting the critical time *t*_*c*_. Picking *t*_*c*_ close to the maximum of the price results in basically the same position of the second peak close to *f* = 1, only the size of the peak changes somewhat.

It is important to mention how our findings relate to actual empirical results. As a benchmark we take the research presented in [43]. It is an interesting observation to note that the series shown in Fig 7A visually very well matches most of the real data based Lomb-periodograms from [43]. The scaling we obtained in the first case is however slightly larger—3.0 compared to 2.0. We expect that varying the parameters *b* and *σ*_*x*_ can influence the log-frequency of the foreshocks. The first parameter governs the periodicity of the bubbles (see Eqs 2 and 3) whereas the second one should be large enough for the foreshocks to appear (as presented in Fig 5), however, we leave detailed testing of this hypothesis for further work. In the second Lomb-periodogram (Fig 7B), one can observe two major peaks—it means that the low frequencies in residuals were not perfectly removed. This series can be compared to the outlying NASDAQ100 in Fig 11 from the aforementioned paper. In fact, the frequency of the peak corresponding to the log-periodicity well matches the data presented in [43]—2.2 versus 2.0.

#### Main crash

The stochastic component modulates the growth of the amplitude of each bubble. Small excursions outwards of a deterministic bubble speed up the evolution and are multiplied by the non-linear forces (see the furthest right part of the deterministic trajectory). This can lead to a rapid crash without any preliminary small corrections. On the other hand, if the trajectory gets pushed inwards by stochastic innovations, it enters the region with a smaller influence from the underlying deterministic dynamics, which can then generate aftershocks.

#### Aftershocks

The aftershocks are purely noisy structures occurring close to the unstable fixed point. The trajectory can wobble around the nullcline or an equilibrium point for an extended period of time. This could be interpreted as a market hesitating on whether to accept that the price has peaked and is due for a correction or a crash, or rather developing some wishful thinking that this is just a temporary consolidation before a new rally starts. Adding noise also in the bond price dynamics can lead to augmenting the variety of consecutive aftershocks.

## Ghosts of finite-time singularities—From derivation to application

This section develops a notion that allows us to provide analytical approximations to the dynamics of stochastically recurrent bubbles described by the SDEs (4). We also provide a guide on how one can use this framework to predict crashes.

### Introductory example

We start with an example explaining what we call *ghosts of finite-time singularities*. We present a study of an ODE known as the *theta model* for a spiking neuron (Eq (3.6) in [44]). We consider the following simplified system with one parameter λ:

$$\begin{array}{c} \dot{\theta }=1-cos\left(\theta \right)+\lambda \;. \end{array}$$ (6)

The solution of the Eq (6) with λ > 0 can be found explicitly, choosing a continuous branch of arctan for:

$$\begin{array}{c} \theta \left(t\right)=2\arctan(\frac{\sqrt{\lambda^{2}+2\lambda }}{\lambda +2}\tan(t\sqrt{\frac{\lambda^{2}}{4}+\frac{\lambda }{2}}+const.)) \end{array}$$ (7)

where the constant term is determined by the initial condition. On the other hand, one can look for the normal form of the saddle-node bifurcation, which is $\dot{x}=x^{2}+\lambda$. Such a form is easily obtained by a Taylor expansion of the cosine function,

$$\begin{array}{c} \cos\left(\theta \right)=1-\frac{\theta^{2}}{2}+O\left(\theta^{4}\right)\;, \end{array}$$ (8)

and the ODE (6) can be approximated by

$$\begin{array}{c} \dot{\theta }=\frac{\theta^{2}}{2}+\lambda \end{array}$$ (9)

with the explicit solution

$$\begin{array}{c} \theta \left(t\right)=\sqrt{2\lambda }\tan(t\sqrt{\frac{\lambda }{2}}+const.)\;. \end{array}$$ (10)

This solution exhibits a singular behaviour for the times *t*_*n*_ such that $t_{n}\sqrt{\lambda /2}+const.=\left(2n+1\right)\pi /2$ becomes an odd multiple of *π*/2. In contrast, the exact solution (7) does not have such a divergence, with *θ* remaining finite at all times. Rather than showing a divergence at these times *t*_*n*_, *θ* jumps from positive to negative. Close to these times *t*_*n*_ but not too close such that $\tan(t\sqrt{\lambda /2}+const.)$ is sufficiently smaller than 1 so that one can expand the arctan to equal its argument, and neglecting terms of order λ^2^ compared to λ and terms of order λ compared to 1, then the exact solution (7) reduces to the solution (10) of the approximating normal form. This shows that the approximate solution shadows the true trajectory very close to the singular times *t*_*n*_. We refer to the jump-like behaviour of the true dynamics as the ghost of the singularity exhibited by the underlying normal form approximating it.

In Eq (10), the constant term plays an important role. If the initial condition in the original system (6) is not directly observable (for instance it might be out of the stable periodic orbit), the constant term in (10) can be used to obtain reliable estimates of the time of the next singularity.

**Fig 8:**
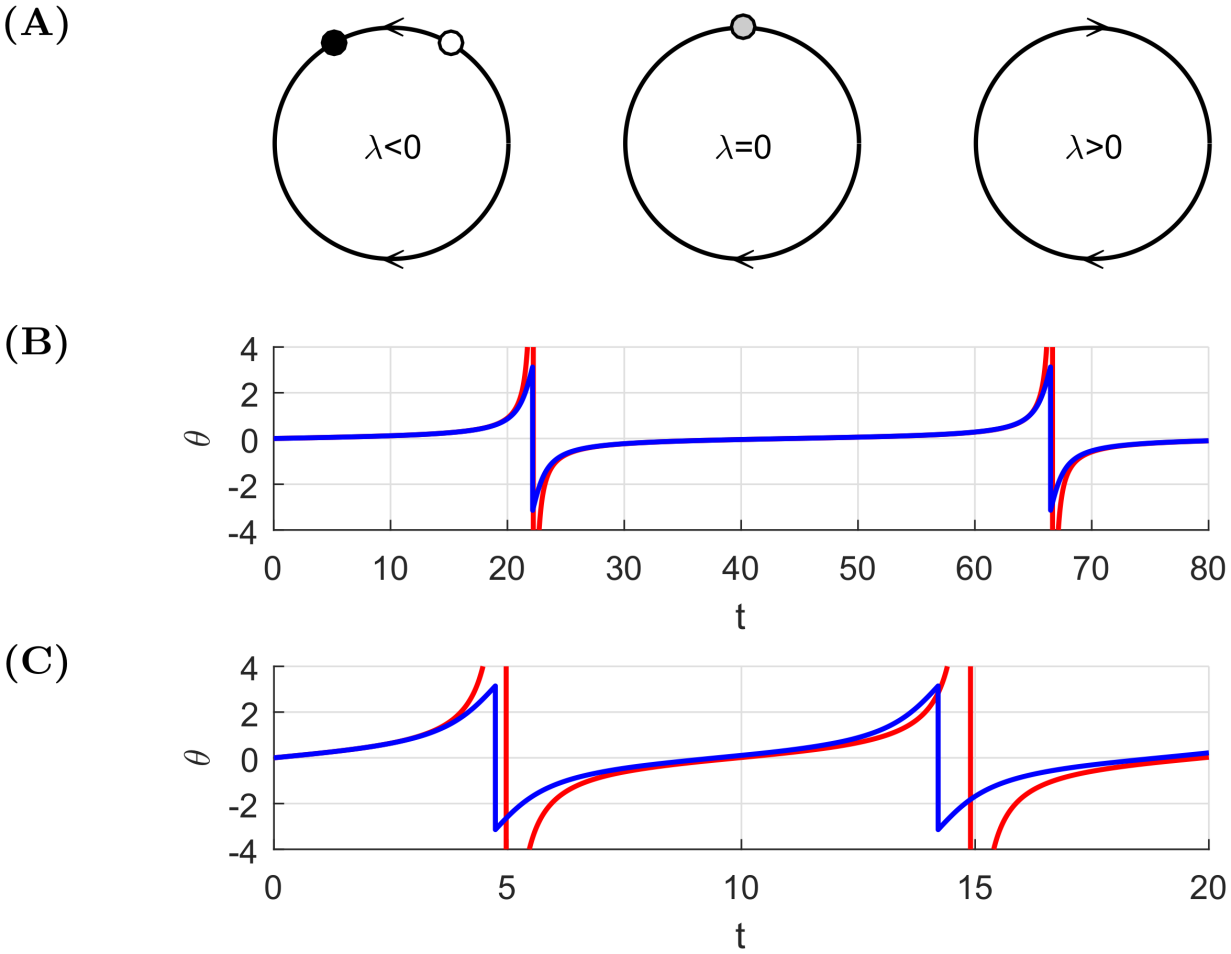
Example of ghosts of finite-time singularities and real finite-time singularities. **(A)** Schematic bifurcation diagram of the flow of system (6). For λ < 0, there are two equilibria—one stable (black) and one unstable (white), for λ = 0, a saddle-node bifurcation occurs and for λ > 0 (if we allow discontinuities in the solutions), one can observe periodic orbits between −*π* and *π*. In the two-dimensional case, discontinuities are not necessary to obtain a periodic behaviour. Panels **(B)** and **(C)** show the sample solutions for systems (6) and (9). The blue curve is the original accurate solution of the full dynamics, where no finite-singularity occurs. The red one is the approximation based on the saddle-node normal form. In the solution based on normal form, there are finite-time singularities whereas, in the original one, bubbles and crashes are bounded. The jump behaviour exhibited by the exact solution of the full system is called the *ghost of the singularity* associated with the normal form approximation. Parameter values are: (B) λ = 0.01 and (C) λ = 0.2.

We assume that λ > 0, hence the system exhibits a periodic behaviour as in Fig 8. From Eqs (7) and (10), it is a simple calculation to obtain the periods, which are not identical due to the differences in the argument of the tangent function. But, the smaller the absolute value of λ, the smaller is the difference in periodicity and the more accurate the predictions obtained from the normal form approximation (9) of the full system (6). We conclude that the approximation of the true solution leads to a good estimation of the period even if its amplitude does not match that of the original system. This result can be used to make predictions on the future state of the system with no need for simulations.

### Analytical derivation of the ghosts of finite-time singularities in the system of stocks and bonds

Building in the insights of the previous section, we now calculate the extended centre manifold of the system (1) in the parameter regime with bubbles and will use it to approximate the solutions of the full system. The detailed analysis is presented in S3 Appendix and leads to the final form

$$\begin{array}{c} {\bar{x}}_{app}\left(t\right)=A+B\tan\left(C\left(t-D\right)\right)+E\tan^{2}\left(C\left(t-D\right)\right)\;, \end{array}$$ (11)

where the higher order term might be dropped, hence for simplicity we also consider expression

$$\begin{array}{c} x_{app}\left(t\right)=A+B\tan\left(C\left(t-D\right)\right)\;. \end{array}$$ (12)

The calculation gives explicitly all parameters but one—*D*. This parameter *D* cannot be computed in the same way as the others, since the (movable) singularities of *x*_*app*_(*t*) are at positions that strictly depend on the initial condition of the system [45]. The approximation suggested by [10], which reads

$$\begin{array}{c} {\hat{x}}_{app}\left(t\right)=\frac{c_{1}}{{\left(t_{\Lambda }-t\right)}^{\beta }}\exp\{\frac{c_{2}}{{\left(t_{\Lambda }-t\right)}^{\alpha }}\}\;, \end{array}$$ (13)

features super-exponential and finite-time singularity properties, just as (11) and (12). The most important achievement coming from all the aforementioned approximations is that they can be used to predict the time of a crash as being at or close to the time of the singularity. For ${\hat{x}}_{app}$ given by (13), it would be directly *t*_Λ_, whereas for ${\bar{x}}_{app}$ and *x*_*app*_, additional calculations are required. Knowing that $\tan(\frac{\pi }{2}+k\pi )=\infty$, the estimated crash time *t*_*c*_ are determined by the condition $C\left(t_{c}-D\right)=\frac{\pi }{2}+k\pi$, which gives

$$\begin{array}{c} t_{c}=\inf\{\frac{\pi /2+k\pi }{C}+D>t_{2}:k\in N\}\;, \end{array}$$ (14)

where *t*_2_ is the end of the given sample used to predict a crash.

**Fig 9:**
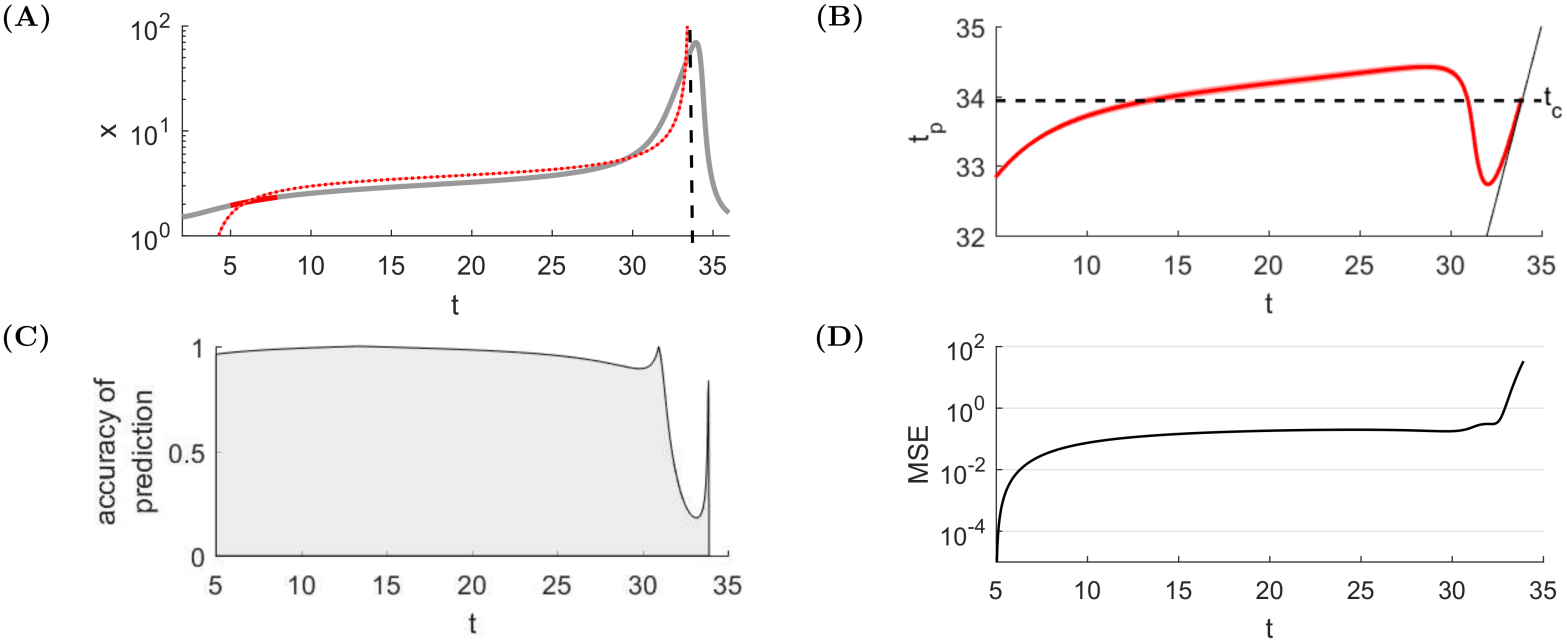
Sample application of the ghosts of finite-time singularities in the deterministic system (1) for parameters *b* = 0.42 and *g* = −0.04. **(A)** Presentation of one fit (thin red line) for a small sample set representing current knowledge up to ‘present time’ *t*_2_ = 8 (bold red) compared with the true price trajectory (grey). The predicted time of the crash (i.e. where the finite-time singularity occurs in (12)—indicated by the dashed black line) is very close to the peak of the bubble and therefore provides an accurate forecast. **(B)** Time *t*_*p*_ of the predicted crash (red) as a function of the ‘present’ time *t* until which we assume we have knowledge of the price dynamics, compared with the exact crash time *t*_*c*_. We apply here a sliding window of arbitrary length *w* = 3. The following section explains the methodology that we follow to choose the optimal window length. When the bubble starts to grow rapidly (around *t* = 30), the predicted crash time *t*_*c*_ decreases towards the present time *t* and, as *t* increases, *t*_*p*_ remains close to *t* as can be seen by the line *t*_*p*_ = *t* (solid black). The price acceleration thus tends to induce the calibration to believe that the crash is looming, exaggerating the imminence of the danger. **(C)** Accuracy of crash prediction measured by 1 − |*t*_*c*_ − *t*_*p*_|/|*t*_*c*_ − *t*_2_|. The prediction accuracy is remarkably high already very far from the crash, and does not improve significantly over most of the lifetime of the bubble. However, when time passes 30, the accuracy deteriorates dramatically. It is caused by the fact that the predictions are based on the normal form truncation close to equilibrium. This is not the case after time 30 when the system grows exponentially. In other words, the prediction system develops a myopic optical illusion when approaching close to the end of the bubble. **(D)** Mean Square Error for the fitted function (12). When the bubble starts to rise rapidly, the MSE increases very quickly, confirming the lost of reliability of the prediction.

In order to provide greater flexibility in the fitting scheme and to approach the fact that the trajectories have a tendency to progress very slowly for low values of *x*, we allow in the fitting procedures vertical correction, i.e. parameter *A* will be fitted as well. Fig 9 illustrates the application of the ghosts of finite-time singularities in the deterministic system (1). It demonstrates that the singular dynamics (12) provides remarkably accurate predictions of the ghost of the singularity, i.e., of the peak of the real bubble dynamics.

### Selection of the optimal window length

Before one applies the above results, there is one more parameter to determine—the window length *w* of the time series to which the function (12) is fitted. It is important to realise that one should not use the whole price history, but only the rather recent one when the price starts to acceleration and the bubble starts to develop. Before that, the price is close to its ghost equilibrium fixed point and is mostly exhibiting a random walk. Moreover, as the function (12) exhibits periodic finite-time singularities, when one wants to predict the next singularity, including the previous one in the analysis will be highly disruptive to the search algorithm (in our case: Levenberg-Marquardt). The simple solution to avoid such situation is to bound the window length, for instance to 70% of the fitted function’s period and to check several initial guesses picking the one with the least mean square error or the smallest predicted time of a crash following the final (i.e. ‘present’) time *t*_2_ of the sample. In our computation, the second criterion is used, as our objective is to predict the singular time *t*_*p*_ of the bubble collapse.

**Fig 10:**
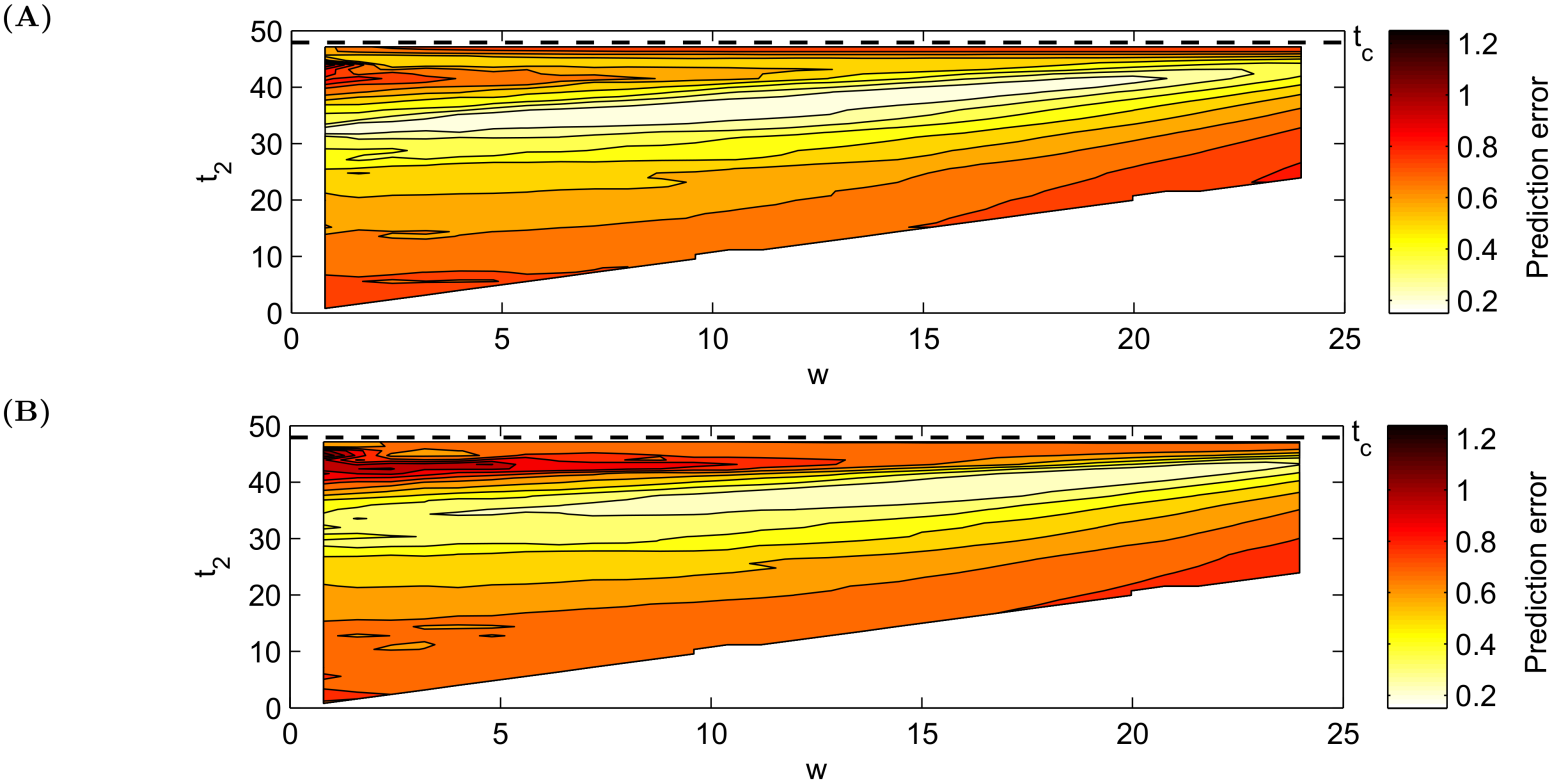
Dependence of the prediction error $\frac{|t_{c}-t_{p}|}{|t_{c}-t_{2}|}$ as a function of window size *w* and end *t*_2_ of window. **(A)** For *σ*_*x*_ = 0.2, the optimal window length is around 10. **(B)** For *σ*_*x*_ = 0.4, the optimal window length is around 15. The parameters of the model are *b* = 0.42, *g* = −0.04, *σ*_*z*_ = 0.01, $\delta (W_{t}^{\left(1\right)},W_{t}^{\left(2\right)})=50\%$. For both selected window sizes, the error just before the time of a crash *t*_*c*_ does not significantly differ in comparison to larger *w*’s and, moreover, the smaller *w* is, the faster the system responds to new information. There is thus a trade-off between responsive adaptation of the fits and error size.

On the other hand, the window length cannot be too small as even a tiny perturbation would cause *t*_*p*_ to vary significantly and the outcome would not be reliable anymore. In order to avoid any a priori bias, we propose to scan both window lengths *w* and end of sample time *t*_2_ on 100 randomly generated bubbles. Afterwards, for each time window, the prediction error is quantified as $\frac{|t_{p}-t_{c}|}{|t_{2}-t_{c}|}$, with the top and bottom 10% cases being put aside to remove outliers and ensure robust results, and the rest is averaged. The procedure maps one prediction error value to each pair (*w*, *t*_2_). Fig 10 presents the final results.

Based on Fig 10, for the following simulations presented in the next section, we will use window size *w* = 10 for *σ*_*x*_ = 0.2 and *w* = 15 for *σ*_*x*_ = 0.4. For these selected values of *w*, the prediction errors are the smallest over the widest range of window size *w* and window ends *t*_2_, making the results robust.

### Application of ghosts of finite-time singularities

**Fig 11:**
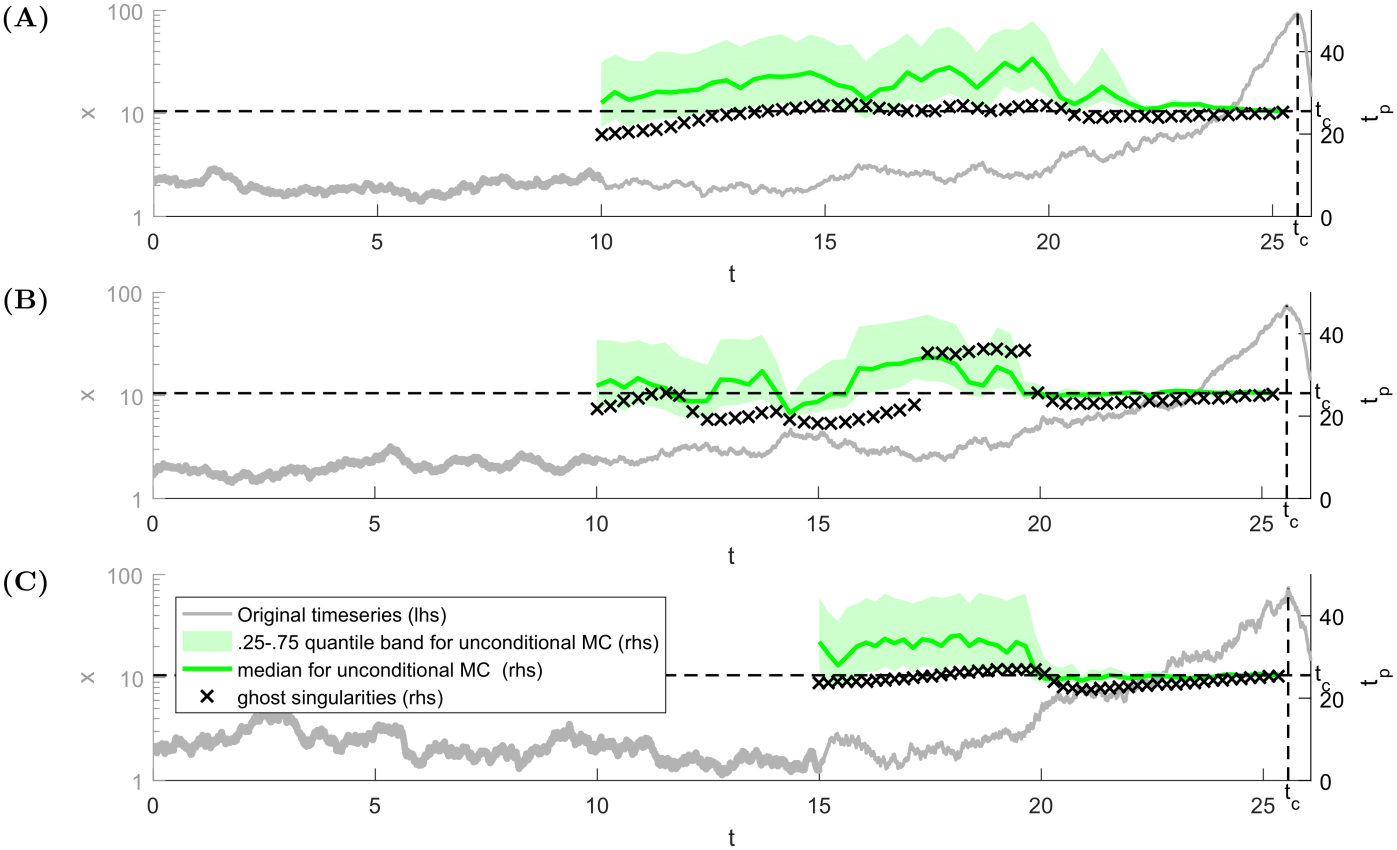
Test of the methodology using the ghosts of finite-time singularities in comparison with Monte Carlo forecasting method. **(A)** and **(B)** correspond to two different realizations of the price process for *σ*_*x*_ = 0.2 and *σ*_*z*_ = 0.01, **(C)** corresponds to one realisation generated with *σ*_*x*_ = 0.4 and *σ*_*z*_ = 0.01. The stochastic price trajectory is plotted (grey line) with its scale given on the left hand side vertical axis. The scale of the crash time predictions is given by the right hand side vertical axis. The symbols ‘x’ stand for the predicted time *t*_*p*_, using the history from time *t* − *w* until time *t* to perform the calibration with the singular model. The criteria used to select the window size *w* for different noise amplitudes are given in the previous section.

When all parameters are estimated, we can finally determine how the methodology of ghosts of finite-time singularities can work in practice. Firstly, we generate a single trajectory from the SDE model (4) with a sufficiently long price history preceding a crash. The variable *x* is shown as the grey line in Fig 11 with its scale given on the left hand side vertical axis. The knowledge of the model parameters, as determined in S3 Appendix, gives explicitly the period and the slope of the tangent function (12) used to approximate the emerging bubbles.

Then, based on the considerations presented in the previous section, the size of the sliding window of analysis is chosen. For *σ*_*x*_ = 0.2, it is *w* = 10, hence the first prediction (or calibration) is done at time *t*_2_ = 10, which allows us to take into account the history in the window *t* ∈ [0, 10]. This initial window is shown as a bold grey line close the horizontal axis in Fig 11. The corresponding predicted crash time *t*_*p*_(*t*_2_ = 10, *w* = 10) is indicated by the first black ‘x’ marker (with its scale given on the right hand side vertical axis). Thereafter, the calibration window is shifted, while keeping its size fixed, which mimics the passing of time as the bubble develops and we accumulate data to perform real-time forecasts, while removing data of the far away past. Implementing this procedure gives us the evolution of *t*_*p*_ as a function of ‘present’ time *t*_2_, offered as a forecast for the real time *t*_*c*_ of the crash. We continue the procedure until *t*_2_ approaches close to the true *t*_*c*_.

The forecasts obtained by using the tangent function (12) are compared with those of the following Monte Carlo scheme using the exact Eq (4). We integrate 500 trajectories with the known exact parameters and initiated in the current state (*x*_*t*_, *z*_*t*_), and record the time $t_{p}^{MC,i}$ (*i* = 1, …, 500) of the first crash that appears in each trajectory. Operationally, we define a crash by the occurrence of a maximum of a bubble that rose above 50% and then collapsed to a value below 20% of the underlying deterministic stable bubble that would exist for the same parameter values. For the selected level of noise, this criterion has been found to be very reliable. Then, the forecast $t_{p}^{MC}$ is the median value of the all crash times $t_{p}^{MC,i}$ over this population of 500 price trajectories (green line in Fig 11). The ensemble $t_{p}^{MC,i}$ (*i* = 1, …, 500) also allows us to give the inter-quartile interval of confidence (the light green band in Fig 11).

To determine the influence of the amplitude of the noise process on the quality of the forecasts, we present two different outcomes for the same noise value *σ*_*x*_ = 0.2 (Fig 11A and 11B) and one for *σ*_*x*_ = 0.4 (Fig 11C). It is noteworthy that these diagrams differ significantly. In the first case (Fig 11A), the price trajectory happens to be very regular, and the predictions are found to be very accurate over a large time interval. For the second price realisation (Fig 11B) that exhibits stochastic foreshocks, the forecasts are more unstable, as a result of the influence of disjoint local attractors in the parameter space. The disappearance of one of them with the lowest *t*_*p*_ leads visually to a discontinuous transition towards a different state around time *t* = 17. On the other hand, the forecasts are definitely closer to the true *t*_*c*_ than the Monte Carlo scheme. One can also note that the forecasts become excellent when *t*_2_ passes the value 20 beyond which the price starts its characteristic bubble acceleration.

For the higher level of noise (Fig 11C), two regimes can be observed. First, *t*_*p*_ steadily increases as a function of *t*_2_. Then, around *t*_2_ = 20, one can observe a quick decreasing phase, which is caused by a sudden escalation of the variable *x*. This suggests that the method of ghosts of finite-time singularities provides a cautious approach as it quickly reacts to variable changes while being on the conservative side with *t*_*p*_ in general smaller than the true *t*_*c*_. In contrast, the Monte Carlo forecast errs towards larger values up to *t*_2_ = 20 and then converges quickly to the correct value.

In summary, it is remarkable that the method of ghosts of singularities provides in general a better forecast than the full integration of the true dynamical stochastic equation. By reducing the complexity and focusing on the key ingredient underlying the forecast skill, namely the time to the bubble, the method of ghosts of singularities seems to be less sensitive to idiosyncratic noise realisations, thus providing more robust forecasts. This can be interpreted as a kind of effective coarse-graining of the dynamical equations, a process that, when done intelligently, has been shown in the past to improve predictability [46–49].

## Discussion and conclusions

Revisiting the non-linear model of coupled stock and bond prices exhibiting periodically collapsing bubbles recently proposed by [10], we have been able to prove and document a number of novel important properties. We have extended the previous analysis of [10] concerning the classification of a rich set of bifurcations in the two-parameter space of this model, organised by codimension-two cusp and Bogdanov-Takens points. We have also confirmed analytically the numerical results concerning the bubble amplitude scaling as 1/|*g*| as a function of the parameter *g* quantifying the sensitivity of the fundamental bond price to past asset prices, when it approaches 0 from below. Moreover, following [33], we have shown that there are two bifurcation paths along which a periodic orbit has its period diverging, which are associated with two different scaling laws: depending on the distance Δ = |*p* − *p*_*bif*_| from the bifurcation line, the period diverges as |ln Δ| for an homoclinic bifurcation or as $\Delta^{-\frac{1}{2}}$ for a saddle-node invariant circle, which are both present in the studied system.

Using a detailed phase space representation and spectral analysis, we have been able to characterise the forces controlling the bubble growth and deflation in the presence of stochastic multiplicative noise. We found that the characteristics of the acceleration of the dynamics in phase space and the shape of the deterministic vector field are two major causes of the price patterns resembling the log-periodic power law singularity structures observed in real financial prices.

Finally, we have provided an analysis to show how dramatic shifts in such a system can be predicted. By expanding the system in the neighbourhood of the saddle-node bifurcation, we obtained a function that approximates an arising bubble. This function exhibits finite-time singularities and, therefore, it cannot be used to predict the precise system state far from the equilibrium trajectory. Nevertheless, its periodicity still matches well that of the original system. This property gives a simple tool to predict when the system is going to crash. We have shown by considering a few realisations of the stochastic price process how the idiosyncratic occurrence of noise innovations and increasing volatility impact the performance of the predictions. We have introduced the notion of ‘ghosts of finite-time singularities’, based on a normal form approximating the true dynamics and which exhibits a finite-time singularity while the true system does not. But it turns out that the time of the peak of the bubble in the true system is very well approximated by the singularity time of the approximating normal form. Hence, the peak of the bubble can be viewed as a kind of ‘ghost’ of the finite-time singularity expressed in the approximating normal form. We have shown that this concept is very useful to predict the time of crashes by estimating the remaining time to the end of an evolving bubble, using the approximated normal form valid close to a bifurcation point. We have tested the forecasting skill of the method of ‘ghosts of finite-time singularities’ on different stochastic price realisations, in comparison with the full integration of the true dynamical stochastic equation. Remarkably, we have found that the former is significantly more precise and less biased than the construction of many scenarios built on the full integration of the exact stochastic differential equations. The mechanism underlying this augmented performance has been argued to result from a reduction of complexity that focuses on the key ingredient underlying the forecast skill, namely a ghost singular behaviour, which leads to a smaller sensitivity to idiosyncratic noise realisations, thus providing more robust forecasts.

## Supporting information

S1 Appendix Codimension-two bifurcations. (PDF)

S2 Appendix Amplitude of a bubble. (PDF)

S3 Appendix Centre manifold expansion for an emerging bubble. (PDF)
