# Supplementary material for: Predicting financial market crashes using ghost singularities
Source: PLoS One. 2018 Mar 29;13(3):e0195265. doi: 10.1371/journal.pone.0195265 (PMC5875899; doi:10.1371/journal.pone.0195265)
Supplement: S1 Appendix — (PDF) [file pone.0195265.s001.pdf]

S1 Appendix. Codimension-two bifurcations. In the section explaining bifurcations of the system given by Eq. (1), we mentioned certain codimension-two bifurcation points. One of them is the Bogdanov-Takens point (further information can be found in Chapter 8.4 of [1]). For the Bogdanov-Takens point, the Jacobian has a double-zero eigenvalue, hence it is possible to derive the analytical coordinates for that point, which are the following:

$$\begin{cases} x^*_{BT} = e^2 \approx 7.389 \\ z^*_{BT} = e^{-\frac{1}{2}} \approx 0.6065 \\ b_{BT} = 2e^{-\frac{3}{2}} \approx 0.4463 \\ g_{BT} = -\frac{1}{2}e^{-2} \approx -0.06767 \end{cases} . \tag{S1.1}$$

The second codimension-two bifurcation point is a cusp (Chapter 8.2 in [1]). At that point, an analytical values of its coordinates are as follows:

$$\begin{cases} x^*_{cusp} = e^{\frac{1+\sqrt{5}}{2}} \approx 5.043 \\ z^*_{cusp} = e^{\frac{\sqrt{5}-3}{2}} \approx 0.6825 \\ b_{cusp} = \frac{1+\sqrt{5}}{2}e^{1-\sqrt{5}} \approx 0.4701 \\ g_{cusp} = \frac{\sqrt{5}-3}{2}e^{-\frac{1+\sqrt{5}}{2}} \approx -0.07574 \end{cases} . \tag{S1.2}$$

References

[1] Kuzntesov YA. Elements of Applied Bifurcation Theory. New York: Springer; 2004.
