# Supplementary material for: Predicting financial market crashes using ghost singularities
Source: PLoS One. 2018 Mar 29;13(3):e0195265. doi: 10.1371/journal.pone.0195265 (PMC5875899; doi:10.1371/journal.pone.0195265)
Supplement: S2 Appendix — (PDF) [file pone.0195265.s002.pdf]

S2 Appendix. Amplitude of a bubble. In order to determine how high can a bubble rise, let us first find a lower bound for the amplitude. Let x_{max} be the maximal value of x and x_{eq} be a non-trivial equilibrium point – both marked in Fig. 1. Obviously, $x_{max} \geqslant x_{eq}$.

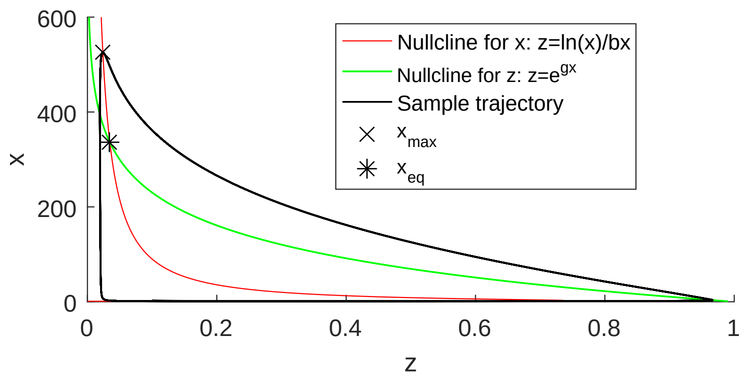

Figure 1: Nullclines for the system (1) with marked x_{max}, x_{eq} and a sample periodic trajectory. Parameters are $b = 0.5$ and $g = -0.01$. As $g \to 0^-$, the nullcline for z shifts to the right, so that the crosspoint with the nullcline for x (the equilibrium point x_{eq}) goes to infinity.

We can obtain the parameter g depending on x_{eq} and b at the crosspoint of the nullclines:

$$\begin{cases} 0 = x_{eq} - x_{eq}^2 \cdot e^{-bx_{eq}z} \\ 0 = z - z^2 \cdot e^{-gx_{eq}} \end{cases} . \tag{S2.1}$$

which yields

$$\begin{cases} x_{eq} = e^{bx_{eq}z} \\ z = e^{gx_{eq}} \end{cases} , \tag{S2.2}$$

and thus

$$|g| = \frac{\ln(bx_{eq}) - \ln(\ln(x_{eq}))}{x_{eq}} . \tag{S2.3}$$

As $x_{eq} \to \infty$, $|g| \to 0^+$.
What is more,

$$\forall \varepsilon > 0 \;\; \exists \hat{x} \;\; \forall x_{eq} > \hat{x} \quad \frac{c_1}{x_{eq}^{1+\varepsilon}} \leqslant |g| \leqslant \frac{c_2}{x_{eq}^{1-\varepsilon}} , \tag{S2.4}$$

where c_1 and c_2 are some positive constants. We will use the notation $|g| \sim \frac{1}{x_{eq}}$ to represent this situation (S2.4).
Translating the previous statement for x_{eq}, we obtain

$$x_{eq} \sim \frac{1}{|g|} . \tag{S2.5}$$

From the numerical calculations presented in Fig. 2, we conclude that

$$\frac{x_{max}}{x_{eq}} \xrightarrow[g \to 0^-]{} c , \tag{S2.6}$$

where $c \in [1, 2)$ is a constant. The data points for $\frac{x_{max}}{x_{eq}}$ shown in Fig. 2 can be fitted very well by the function

$$\frac{x_{max}}{x_{eq}}(g) = \frac{1}{(-\log_{10}|g|)^{\alpha}} + 1\,, \tag{S2.7}$$

where for $\alpha = 0.8328$, $R^2 = 0.9991$. It implies that $c = 1$. From Eq. (S2.5) and Eq. (S2.7), we deduce that

$$x_{max} = \frac{x_{max}}{x_{eq}} \cdot x_{eq} \sim 1 \cdot \frac{1}{|g|} = \frac{1}{|g|}\,. \tag{S2.8}$$

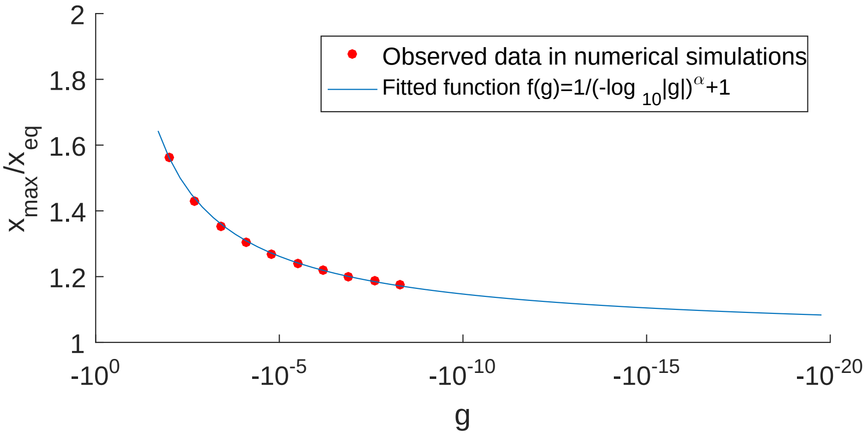

Figure 2: Change of the ratio of the top of bubble to the non-trivial equilibrium point depending on g. Here $b = 0.5$. One can notice extremely good approximation of fitted curve with $R^2 = 0.9991$.
