# Supplementary material for: Predicting financial market crashes using ghost singularities
Source: PLoS One. 2018 Mar 29;13(3):e0195265. doi: 10.1371/journal.pone.0195265 (PMC5875899; doi:10.1371/journal.pone.0195265)
Supplement: S3 Appendix — (PDF) [file pone.0195265.s003.pdf]

S3 Appendix. Centre manifold expansion for an emerging bubble. This section provides analytical derivation of a bubble in the vicinity of a saddle-node bifurcation. In order to find a good approximate function of the price dynamics $x(t)$ of a bubble, we calculate $x(t)$ for parameters close to the upper saddle-node branch shown in Fig 2A. On the upper part of the saddle-node curve, let us take consider parameters (b^*, g^*) and the corresponding non-trivial equilibrium point (x^*, z^*). We fix b^* and vary only parameter g, so instead of (1), we consider the following system:

$$\begin{cases} \dot{x} = x - x^2 e^{-b^* xz} \\ \dot{z} = z - z^2 e^{-(g^*+\delta)x} \,. \end{cases} \tag{S3.1}$$

Next, we move the system to the origin so that the equilibrium point is placed at $(0, 0)$:

$$\begin{cases} X = x - x^* \\ Z = z - z^* \\ b = b^* \\ g = g^* + \delta \end{cases} \tag{S3.2}$$

and, by taking into account the derivative $\frac{d\delta}{dt}$, we obtain

$$\begin{pmatrix} \dot{X} \\ \dot{Z} \\ \dot{\delta} \end{pmatrix} = \begin{pmatrix} (X + x^*) - (X + x^*)^2 e^{-b^*(X+x^*)(Z+z^*)} \\ (Z + z^*) - (Z + z^*)^2 e^{-(g^*+\delta)(X+x^*)} \\ 0 \end{pmatrix} = \begin{pmatrix} F(X, Z, \delta) \\ G(X, Z, \delta) \\ 0 \end{pmatrix} . \tag{S3.3}$$

The Jacobian in the equilibrium point is

$$J := J(X, Z, \delta)\Big|_{(0,0,0)} = \begin{pmatrix} \frac{\partial F}{\partial X} & \frac{\partial F}{\partial Z} & 0 \\ \frac{\partial G}{\partial X} & \frac{\partial G}{\partial Z} & \frac{\partial G}{\partial \delta} \\ 0 & 0 & 0 \end{pmatrix}_{(0,0,0)} \overset{(*)}{=} \begin{pmatrix} p_1 & p_2 & 0 \\ p_3 & \frac{p_2 p_3}{p_1} & p_4 \\ 0 & 0 & 0 \end{pmatrix} , \tag{S3.4}$$

where the substitution $(*)$ is performed in order to simplify the notations for the saddle-node equilibrium point where the Jacobian $\begin{pmatrix} \frac{\partial F}{\partial X} & \frac{\partial F}{\partial Z} \\ \frac{\partial G}{\partial X} & \frac{\partial G}{\partial Z} \end{pmatrix}$ is singular. J has eigenvalues $(\lambda_c, \lambda_s, \lambda_c) = (0, \frac{p_1^2 + p_2 p_3}{p_1}, 0)$ and the eigenvectors for the two first eigenvalues are

$$v_{c_1} = \begin{pmatrix} -\frac{p_2}{p_1} \\ 1 \\ 0 \end{pmatrix} \quad \text{and} \quad v_s = \begin{pmatrix} \frac{p_1}{p_3} \\ 1 \\ 0 \end{pmatrix} . \tag{S3.5}$$

For the third eigenvalue λ_c, we need to find the generalized eigenvector v_{c_2}

$$J v_{c_2} = v_{c_1} \quad \Longrightarrow \quad J^2 v_{c_2} = J v_{c_1} = 0 \,, \tag{S3.6}$$

hence, we calculate the eigenvectors of J^2 for the eigenvalue λ_c. One is of course v_{c_1} and

the other is

$$v_{c_2} = \begin{pmatrix} -\frac{p_2 p_4}{p_1^2+p_2p_3} \\ 0 \\ 1 \end{pmatrix} . \tag{S3.7}$$

In the vector basis $P := (v_{c_1}, v_s, v_{c_2})$, new coordinates can be obtained by the following transformation

$$\begin{pmatrix} X \\ Z \\ \delta \end{pmatrix} = P \begin{pmatrix} U \\ V \\ \delta \end{pmatrix} = \begin{pmatrix} -\frac{p_2}{p_1} & \frac{p_1}{p_3} & -\frac{p_2p_4}{p_1^2+p_2p_3} \\ 1 & 1 & 0 \\ 0 & 0 & 1 \end{pmatrix} \begin{pmatrix} U \\ V \\ \delta \end{pmatrix} \tag{S3.8}$$

and on the other hand

$$\begin{pmatrix} U \\ V \\ \delta \end{pmatrix} = P^{-1} \begin{pmatrix} X \\ Z \\ \delta \end{pmatrix} = \begin{pmatrix} -\frac{p_1p_3}{p_1^2+p_2p_3} & \frac{p_1^2}{p_1^2+p_2p_3} & -\frac{p_1p_2p_3p_4}{(p_1^2+p_2p_3)^2} \\ \frac{p_1p_3}{p_1^2+p_2p_3} & \frac{p_2p_3}{p_1^2+p_2p_3} & -\frac{p_1p_2p_3p_4}{(p_1^2+p_2p_3)^2} \\ 0 & 0 & 1 \end{pmatrix} \begin{pmatrix} X \\ Z \\ \delta \end{pmatrix} . \tag{S3.9}$$

Hence, in the new coordinates, the dynamical system becomes

$$\begin{pmatrix} \dot{U} \\ \dot{V} \\ \dot{\delta} \end{pmatrix} = \begin{pmatrix} \frac{dU(X,Z)}{dt} \\ \frac{dV(X,Z)}{dt} \\ 0 \end{pmatrix} = \begin{pmatrix} (P^{-1})_{11}\dot{X} + (P^{-1})_{12}\dot{Z} \\ (P^{-1})_{21}\dot{X} + (P^{-1})_{22}\dot{Z} \\ 0 \end{pmatrix} . \tag{S3.10}$$

Using the multivariate Taylor expansion for $\dot{U}$, $\dot{V}$ and $\dot{\delta}$ at the point $(0,0,0)$, we obtain

$$\begin{aligned} \begin{pmatrix} \dot{U} \\ \dot{V} \\ \dot{\delta} \end{pmatrix} &= \begin{pmatrix} 0 & 0 & \mu_1 \\ 0 & \mu_2 & 0 \\ 0 & 0 & 0 \end{pmatrix} \begin{pmatrix} U \\ V \\ \delta \end{pmatrix} + \\ &+ \begin{pmatrix} a_1U^2 + a_2UV + a_3U\delta + a_4V^2 + a_5V\delta + a_6\delta^2 \\ b_1U^2 + b_2UV + b_3U\delta + b_4V^2 + b_5V\delta + b_6\delta^2 \\ 0 \end{pmatrix} , \end{aligned} \tag{S3.11}$$

where the coefficients μ_i (for $i \in \{1,2\}$), a_i and b_i (for $i \in \{1,...,6\}$) are known. It is important to mention that the Jacobian of the system (S3.11) has two vanishing eigenvalues and forms a Jordan normal form with separated centre (U_c, δ_c) and stable (V_s) parts.

The flow on the stable manifold can be approximated by

$$V_s(U_c, \delta_c) = \alpha U_c^2 + \beta U_c \delta_c + \gamma \delta_c^2 , \tag{S3.12}$$

hence

$$\dot{V}_s(U_c, \delta_c) = \frac{\partial V_s}{\partial U_c}\dot{U}_c + \frac{\partial V_s}{\partial \delta_c}\dot{\delta}_c = (2\alpha U_c + \beta\delta_c)\dot{U}_c . \tag{S3.13}$$

In order to determine α, β and γ we need to compare coefficients in $\dot{V}_s(U_c, \delta_c)$ and $\dot{V}(U_c, \delta_c)$:

$$
\begin{aligned}
&\mu_2 V_s + b_1 U_c^2 + b_2 U_c V_s + b_3 U_c \delta_c + b_4 V_s^2 + b_5 V_s \delta_c + b_6 \delta_c^2 = \\
&= (2\alpha U_c + \beta \delta_c)(\mu_1 \delta_c + a_1 U_c^2 + a_2 U_c V_s + a_3 U_c \delta_c + a_4 V_s^2 + a_5 V_s \delta_c + a_6 \delta_c^2)
\end{aligned} \tag{S3.14}
$$

When inserting (S3.12) into (S3.14), it is enough to compare the coefficients up to quadratic terms:

$$
\begin{aligned}
\boldsymbol{U_c^2}: &\quad \mu_2 \alpha + b_1 = 0 &\implies& \quad \alpha = -\frac{b_1}{\mu_2} \\
\boldsymbol{U_c \delta_c}: &\quad \mu_2 \beta + b_3 = 2\alpha\mu_1 &\implies& \quad \beta = -\frac{2b_1\mu_1 + b_3\mu_2}{\mu_2^2} \\
\boldsymbol{\delta_c^2}: &\quad \mu_2 \gamma + b_6 = \beta\mu_1 &\implies& \quad \gamma = -\frac{2b_1\mu_1^2 + b_3\mu_1\mu_2 + b_6\mu_2^2}{\mu_2^3}\,.
\end{aligned} \tag{S3.15}
$$

Finally, in order to obtain the flow on the centre manifold, we insert (S3.12) with determined coefficients (S3.15) into $\dot{U}$ (S3.11):

$$
\begin{aligned}
\dot{U}_c = f(U_c, \delta_c) &= \mu_1 \delta_c + a_1 U_c^2 + a_2 U_c (\alpha U_c^2 + \beta U_c \delta_c + \gamma \delta_c^2) + \\
&+ a_3 U_c \delta_c + a_4 (\alpha U_c^2 + \beta U_c \delta_c + \gamma \delta_c^2)^2 + a_5 (\alpha U_c^2 + \beta U_c \delta_c + \gamma \delta_c^2)\delta_c + a_6 \delta_c^2 = \\
&= \mu_1 \delta_c + a_6 \delta_c^2 + \gamma a_5 \delta_c^3 + \gamma^2 a_4 \delta_c^4 + U_c (a_3 \delta_c + a_2 \gamma \delta_c^2 + a_5 \beta \delta_c^2 + 2a_4 \beta\gamma\delta_c^3) + \\
&+ U_c^2 (a_1 + a_2 \beta \delta_c + a_5 \alpha \delta_c + 2a_4 \alpha\gamma \delta_c^2 + a_4 \beta^2 \delta_c^2) + U_c^3 (a_2 \alpha + 2a_4 \alpha\beta\delta_c) + U_c^4 a_4 \alpha^2 \quad .
\end{aligned} \tag{S3.16}
$$

In order to approximate the function describing the time dependence of the price of an arising bubble, we integrate $\dot{U}_c$:

$$
\frac{dU_c}{dt} = f(U_c, \delta_c) \quad \implies \quad \frac{dU_c}{f(U_c, \delta_c)} = dt \quad \implies \int \frac{1}{f(U_c, \delta_c)} dU_c = \int dt + const. \tag{S3.17}
$$

hence

$$
\int \frac{1}{f(U_c, \delta_c)} dU_c = t + const.\,. \tag{S3.18}
$$

Without dropping higher order terms, it might not be possible to obtain U_c explicitly, hence we assume, that $\delta_c = \varepsilon$ and as U_c is expected to vary faster than the parameter, we take $U_c = U_{c0}\sqrt{\varepsilon} + O(\varepsilon)$. Then, for the function f, we truncate all terms of order higher than $O(\varepsilon)$, hence $f(U_c, \delta_c) \approx \mu_1 \delta_c + a_1 U_c^2$. From equation (S3.18), using the simplified form of f, we obtain

$$
\frac{1}{\sqrt{a_1 \mu_1 \delta_c}} \arctan\left(\sqrt{\frac{a_1}{\mu_1 \delta_c}} U_c\right) = t + const.\,, \tag{S3.19}
$$

which gives

$$
U_c = \sqrt{\frac{\mu_1 \delta_c}{a_1}} \tan\left(t\sqrt{a_1 \mu_1 \delta_c} + const.\right)\,. \tag{S3.20}
$$

Inserting (S3.20) into (S3.12) leads to

$$V_s = \alpha U_c^2 + \beta U_c \delta_c + \gamma \delta_c^2 = \alpha \left(\sqrt{\frac{\mu_1 \delta_c}{a_1}} \tan\left(t\sqrt{a_1 \mu_1 \delta_c} + const. \right) \right)^2 +$$
$$+ \beta \left(\sqrt{\frac{\mu_1 \delta_c}{a_1}} \tan\left(t\sqrt{a_1 \mu_1 \delta_c} + const. \right) \right) \delta_c + \gamma \delta_c^2 =$$
$$= \gamma \delta_c^2 + \beta \delta_c^{\frac{3}{2}} \sqrt{\frac{\mu_1}{a_1}} \tan\left(t\sqrt{a_1 \mu_1 \delta_c} + const. \right) + \alpha \delta_c \frac{\mu_1}{a_1} \tan^2\left(t\sqrt{a_1 \mu_1 \delta_c} + const. \right) . \tag{S3.21}$$

Then, from (S3.8), (S3.20) and (S3.21) one obtains

$$X = -\frac{p_2}{p_1} U_c + \frac{p_1}{p_3} V_s - \frac{p_2 p_4}{p_1^2 + p_2 p_3} \delta_c = \frac{p_1}{p_3} \gamma \delta_c^2 - \frac{p_2 p_4}{p_1^2 + p_2 p_3} \delta_c +$$
$$+ \left(\frac{p_1 \beta \sqrt{\mu_1} \delta_c^{\frac{3}{2}}}{p_3 \sqrt{a_1}} - \frac{p_2 \sqrt{\mu_1 \delta_c}}{p_1 \sqrt{a_1}} \right) \tan\left(t\sqrt{a_1 \mu_1 \delta_c} + const. \right) + \tag{S3.22}$$
$$+ \frac{p_1 \alpha \mu_1 \delta_c}{p_3 a_1} \tan^2\left(t\sqrt{a_1 \mu_1 \delta_c} + const. \right) ,$$

where all parameters in the above expression are known. Finally, we need to shift back X by the position of the equilibrium according to (S3.2): $x = X + x^*$. One can write the final result in the following simplified form

$$x = A + B \cdot \tan(C(t - D)) + E \cdot \tan^2(C(t - D)) , \tag{S3.23}$$

for which the numerical values of the parameters are presented in Table 1. It is worth mentioning that parameter D, which determines the initial value of the price, can be determined by aligning the inflection point of (S3.23) with the inflection point of the numerical solution. It turns out that the fit performed with the final formula (S3.23) does not decrease the MSE (mean-square error) drastically, unless the formula is corrected by a vertical shift. In Table 1, A^* plays the role, in the shifted version, of parameter A in the final formula (S3.23). The shifted curve matches the numerical solution very well, as it can be seen in Fig 1. Nevertheless, we have not found any justification for that correcting procedure.

Table 1: Parameters for the best fits of the functions family (S3.23) to the numerical solutions. Parameter A^* replaces A as explained in the text, which results in an extremely good alignment in the vicinity of the saddle-node equilibrium, as shown in Fig 1.

b	g	A	A^*	B	C	D	E
0.4	−0.029	3.042	2.977	0.1744	0.02637	62.03	$-0.5416 \cdot 10^{-3}$
0.38	−0.0117	2.819	2.812	0.03683	0.006325	251.8	$-0.8509e \cdot 10^{-5}$

The final formula (S3.23) provides a good fit in the neighbourhood of the equilibrium point. However, far from the equilibrium state, this function gives a sharper slope than

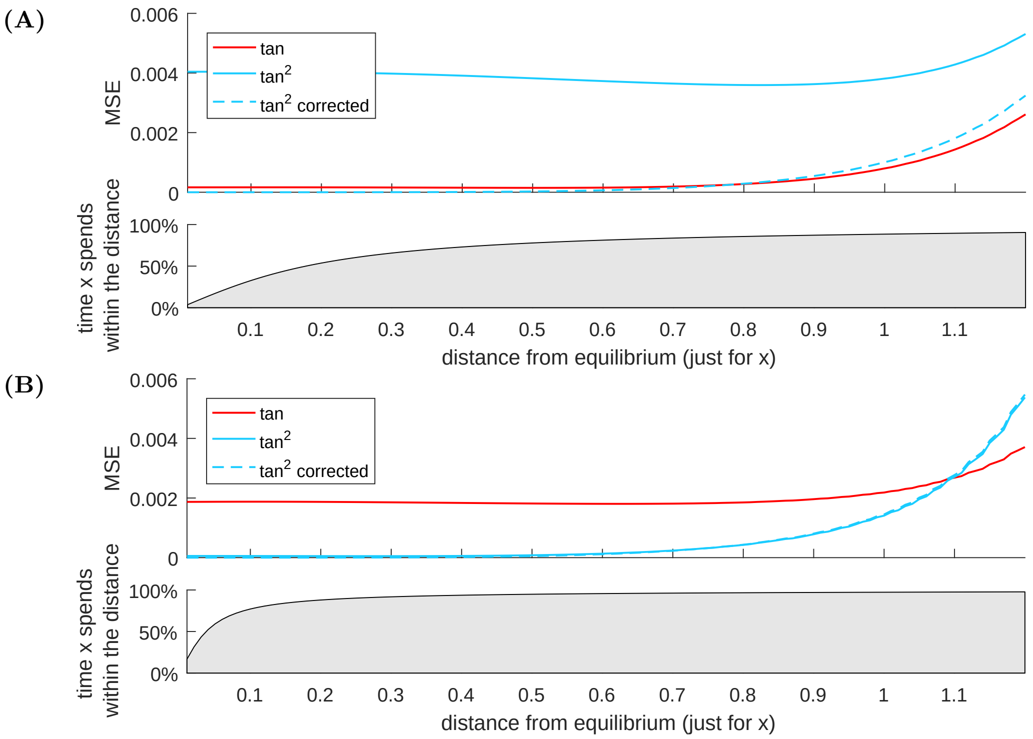

Figure 1: Comparison of the MSE (mean square errors) for (S3.23) and (S3.24). The comparison is presented as a function of the distance from the saddle-node equilibrium, with the percentage of time that the asset price spends within that distance for **(A)** $b = 0.4$, $g = -0.029$ and **(B)** $b = 0.38$, $g = -0.0117$. From the diagram one can deduce that application of vertical correction can decrease the error of the fitted function significantly in some cases but not always. For more rapid bubbles in panel A it would be strongly advised. The percentage of time that the asset price spends within a certain distance (bottom panels of A and B) suggest that the system resides very far from an equilibrium only during a very short period. On the other hand, this period is extremely important as it is the time when the bubbles arise and collapse. Comparison of the blue and red curves implies that even though close to the equilibrium $\tan^2$ gives small MSE, further on it is outperformed by the simplified function (tan) and that function with a vertical correction is used for the predictions presented in the main part of the text.

the following function obtained by removing the higher order term

$$x = A + B \cdot \tan(C(t - D)), \tag{S3.24}$$

which we have also tested. The parameters for the form (S3.24) are obtained using `GraphPad Prism 7` (Nonlinear Regression; least squares fitting method; quantification of goodness-of-fit based on R square) and are presented in Table 2. Parameters B and C are obtained directly from the Eq. (S3.22), only A and D need to be found by curve fitting. The initial value of A is chosen to be close to the value where the stock price spends the most time, whereas the initial value of D should cause that the singularity of tangent function would be close to the crash observed in the trajectory. The fits and the trajectories are presented in Fig 2 in linear scale and Fig 3 in logarithmic scale. In equation $\dot{x} = x - x^2 e^{-bxz}$, for large x, the second component disappears and the slope

becomes exponential. It cannot be observed in neither (S3.22) nor (S3.24), but suggests that, without the force that drags the bubble down to a crash, the acceleration might end in finite-time singularity.

Table 2: Parameters for the best fit of the functions family (S3.24) to numerical solutions with the 95% confidence intervals for parameters A and D. The parameters B and C are obtained in (S3.22). They are $B = 0.1744$ and $C = 0.02637$ for the Figs 2A and 2B and $B = 0.03683$ and $C = 0.006325$ for the Figs 2C and 2D.

Figure	A	95% CI of A	D	95% CI of D
2A	2.991	$(2.630, 3.351)$	62.07	$(62.01, 62.13)$
2B	3.217	$(3.104, 3.329)$	61.78	$(61.76, 61.79)$
2C	2.855	$(2.756, 2.953)$	251.3	$(251.3, 251.4)$
2D	6.031	$(4.799, 7.262)$	251.3	$(251.3, 251.4)$

Comparison of numerical results for the approximate description of the price dynamics of a bubble.

Both the tangent function (S3.24) and the formula (S3.23) with the additional quadratic term seem to provide good fits to the bubble price at least sufficiently close to the equilibrium. We compare them by calculating the mean square error close to the original saddle-node equilibrium, which is reported in Fig 1. One can see that formula (S3.23) gives a very small error close to the equilibrium point, but the error increases much faster than for the tangent function (S3.24) when going away from it. This means that, in order to approximate the trajectory it is better to use (S3.23) close to the equilibrium point and then switch to (S3.24) further away.

References

[1] Yukalov VI, Yukalova EP, Sornette D. Dynamical system theory of periodically collapsing bubbles. Eur Phys J B. 2015;88(7):179–213. doi:10.1140/epjb/e2015-60313-1.

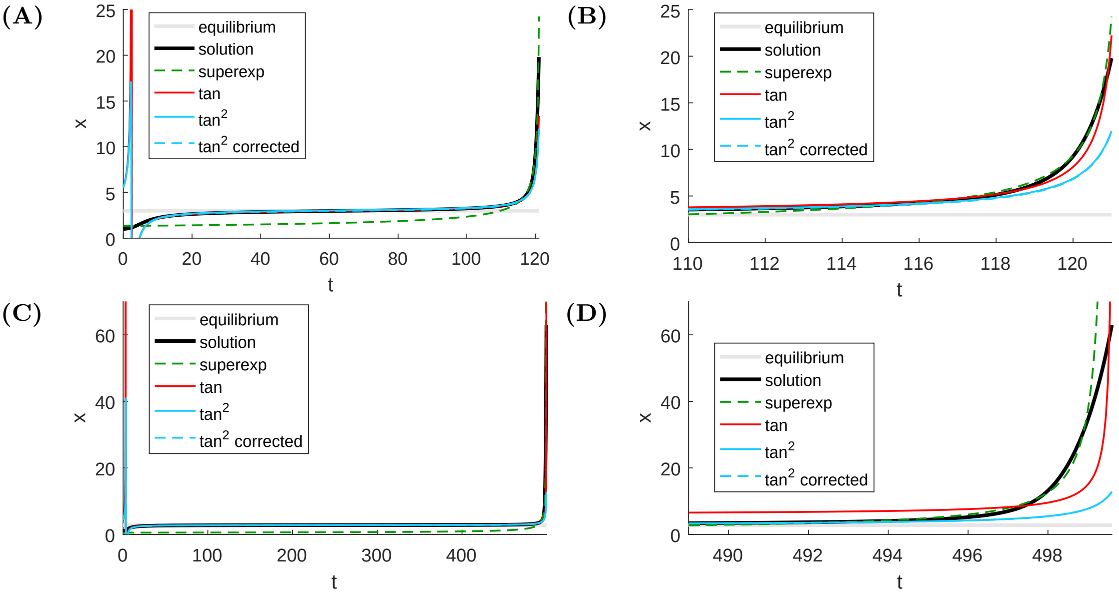

Figure 2: Graphical comparison of the super-exponential fit performed in [1] (see Fig 13 therein) to the fitted functions (S3.23), (S3.24) and (S3.24) with vertical correction. (A) $b = 0.4$, $g = -0.029$, $t \in [0, 121]$, **(B)** $b = 0.4$, $g = -0.029$, $t \in [110, 121]$, **(C)** $b = 0.38$, $g = -0.0117$, $t \in [0, 499.6]$, **(D)** $b = 0.38$, $g = -0.0117$, $t \in [489, 499.6]$.

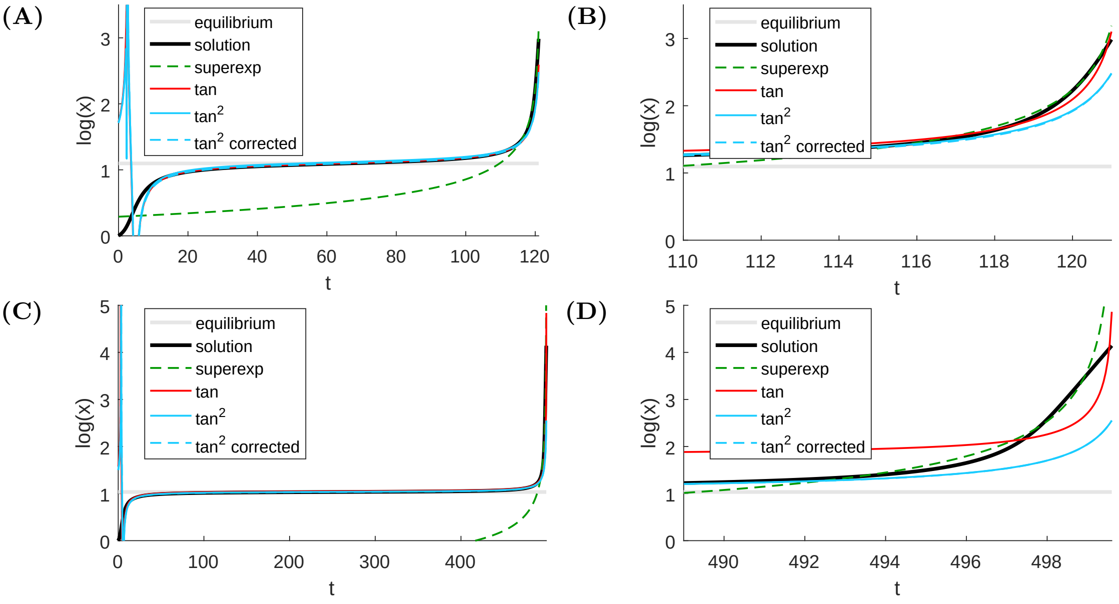

Figure 3: Graphical comparison of logarithm of the super-exponential fit performed in [1] (see Fig 13 therein) to the logarithm of the fitted functions (S3.23), (S3.24) and (S3.24) with vertical correction. (A) $b = 0.4$, $g = -0.029$, $t \in [0, 121]$, **(B)** $b = 0.4$, $g = -0.029$, $t \in [110, 121]$, **(C)** $b = 0.38$, $g = -0.0117$, $t \in [0, 499.6]$, **(D)** $b = 0.38$, $g = -0.0117$, $t \in [489, 499.6]$.
